# Glucagon‐like peptide‐1 ameliorates cardiac lipotoxicity in diabetic cardiomyopathy via the PPARα pathway

**DOI:** 10.1111/acel.12763

**Published:** 2018-04-16

**Authors:** Lujin Wu, Ke Wang, Wei Wang, Zheng Wen, Peihua Wang, Lei Liu, Dao Wen Wang

**Affiliations:** ^1^ Division of Cardiology Department of Internal Medicine Tongji Hospital Tongji Medical College Huazhong University of Science and Technology Wuhan China; ^2^ Hubei Key Laboratory of Genetics and Molecular Mechanism of Cardiologic Disorders Wuhan China; ^3^ Department of Neonatal Medicine The Central Hospital of Wuhan Tongji Medical College Huazhong University of Science and Technology Wuhan China

**Keywords:** apoptosis, DPP‐4, GLP‐1, lipotoxicity cardiomyopathy, PPARα

## Abstract

Lipotoxicity cardiomyopathy is the result of excessive accumulation and oxidation of toxic lipids in the heart. It is a major threat to patients with diabetes. Glucagon‐like peptide‐1 (GLP‐1) has aroused considerable interest as a novel therapeutic target for diabetes mellitus because it stimulates insulin secretion. Here, we investigated the effects and mechanisms of the GLP‐1 analog exendin‐4 and the dipeptidyl peptidase‐4 inhibitor saxagliptin on cardiac lipid metabolism in diabetic mice (DM). The increased myocardial lipid accumulation, oxidative stress, apoptosis, and cardiac remodeling and dysfunction induced in DM by low streptozotocin doses and high‐fat diets were significantly reversed by exendin‐4 and saxagliptin treatments for 8 weeks. We found that exendin‐4 inhibited abnormal activation of the (PPARα)‐CD36 pathway by stimulating protein kinase A (PKA) but suppressing the Rho‐associated protein kinase (ROCK) pathway in DM hearts, palmitic acid (PA)‐treated rat h9c2 cardiomyocytes (CMs), and isolated adult mouse CMs. Cardioprotection in DM mediated by exendin‐4 was abolished by combination therapy with the PPARα agonist wy‐14643 but mimicked by PPARα gene deficiency. Therefore, the PPARα pathway accounted for the effects of exendin‐4. This conclusion was confirmed in cardiac‐restricted overexpression of PPARα mediated by adeno‐associated virus serotype‐9 containing a cardiac troponin T promoter. Our results provide the first direct evidence that GLP‐1 protects cardiac function by inhibiting the ROCK/PPARα pathway, thereby ameliorating lipotoxicity in diabetic cardiomyopathy.

## 1 INTRODUCTION

Diabetes mellitus is expected to reach pandemic proportions over the next few decades. The World Health Organization (WHO) estimates that there will be more than 550 million cases by 2030. These patients are predisposed to cardiovascular morbidity and mortality (Rathmann & Giani, 2004). Cardiovascular events account for two‐thirds of the mortality in patients with diabetes (Boudina & Abel, 2007). Diabetic cardiomyopathy (DCM) is a major cardiovascular complication (Bernardi, Michelli, Zuolo, Candido & Fabris, 2016). DCM is defined as structural and functional myocardial impairments in diabetic patients without coronary artery disease or hypertension. It is mainly characterized by myocardial hypertrophy and fibrosis, metabolic dysregulation, and defects in myocardial contractile properties (Liu et al., 2001). Considerable progress has been made in DCM management. Nevertheless, the molecular etiologies of DCM remain poorly understood, and currently available therapies are far from ideal. Therefore, further research in this area is urgently required.

It has been confirmed that disturbances in cardiac substrate metabolism and energetics are the key contributors to DCM (Anderson et al., 2009; Lopaschuk, Folmes & Stanley, 2007). In diabetes, cardiac palmitate oxidation doubles and glucose oxidation decreases by 30%–40% relative to the levels observed in nondiabetic patients (Anderson et al., 2009; Rijzewijk et al., 2009). Although the switching of substrate utilization may meet the energy demand for heart function maintenance, it also brings many deleterious consequences (Rodrigues, Cam & McNeill, 1995; Stanley, Lopaschuk & McCormack, 1997). Increased fatty acid (FA) oxidation along with reduced ATP/O ratios decreases cardiac efficiency and contributes to ventricular dysfunction by increasing the generation of reactive oxygen species (ROS) and toxic lipid intermediates (Battiprolu et al., 2013; Houstis, Rosen & Lander, 2006). ROS damage DNA, mitochondria, and other cellular components by oxidizing proteins, converting lipids into reactive lipid peroxides, and increasing protein tyrosine nitration (Boudina et al., 2005). Lipid metabolite accumulation in cardiomyocytes (CMs) results in lipotoxicity and apoptosis (Drosatos & Schulze, 2013; van de Weijer, Schrauwen‐Hinderling & Schrauwen, 2011). Therefore, inhibition of FA accumulation and oxidation has become important therapeutic strategies in DCM management.

Peroxisome proliferator‐activated receptor alpha (PPARα) plays an important role in myocardial substrate metabolism by regulating the transcription of genes involved in FA transport, esterification, and oxidation (Banke et al., 2010; Gilde et al., 2003). Increases in FA oxidation and uptake in diabetic hearts were significantly reduced in PPARα^−/−^ mice (Campbell et al., 2002). Cardiac‐restricted PPARα overexpression (MHC‐PPARα) in mice mimicked the DCM phenotype. These animals were relatively more susceptible to serious cardiomyopathy in response to high‐fat diets (HFD) or streptozotocin (STZ) stimulation and presented with significant increases in lipids accumulation (Finck et al., 2002, 2003; Yang et al., 2007). Therefore, PPARα activation‐induced metabolic abnormalities in diabetic hearts may be promising as therapeutic DCM targets.

Native glucagon‐like peptide‐1 (GLP‐1) is a hormone produced by the L‐cells of the distal ileum and colon in response to the entry of nutrients and destroyed by the circulating dipeptidyl peptidase‐4 (DPP‐4) (Ban et al., 2008). In the past decade, GLP‐1 and its analogs have been introduced as a new class of antidiabetic medications for their pleiotropic effects, including increasing glucose‐dependent insulin secretion, suppressing glucagon secretion, decreasing appetite, and reducing body weight (Park, Lim, Lee & Na, 2016). A functional GLP‐1 receptor (GLP‐1R) is highly expressed in the heart. GLP‐1R agonists and DPP‐4 inhibitors have beneficial effects on the cardiovascular system (Ban et al., 2008; Noyan‐Ashraf et al., 2009; Timmers et al., 2009). Previous studies have shown that GLP‐1 and its analogs protected the heart against ischemia‐reperfusion injury and diabetes mellitus (Tate, Robinson, Green, McDermott & Grieve, 2016; Wang et al., 2013). They also protected isolated CMs from oxidative damage (Chang et al., 2013) and high‐glucose stress (Younce, Burmeister & Ayala, 2013). However, the protective effects of GLP‐1 exendin‐4 (Ex‐4) on lipid metabolism in diabetic hearts and the relationship between GLP‐1R activation and the PPARα pathway have not yet been elucidated.

In this study, we investigated the effects of the GLP‐1R agonist exendin‐4 and the DPP‐4 inhibitor saxagliptin on DCM induced by a HFD and STZ injections. We demonstrated that the cardioprotective effects of GLP‐1, including reductions in lipid accumulation and potentiation of antioxidant and anti‐apoptosis properties, may be driven by a PPARα‐mediated mechanism.

## 2 RESULTS

### 2.1 Exendin‐4 and saxagliptin reversed symptoms in diabetic mice (DM)

**Figure 1:**
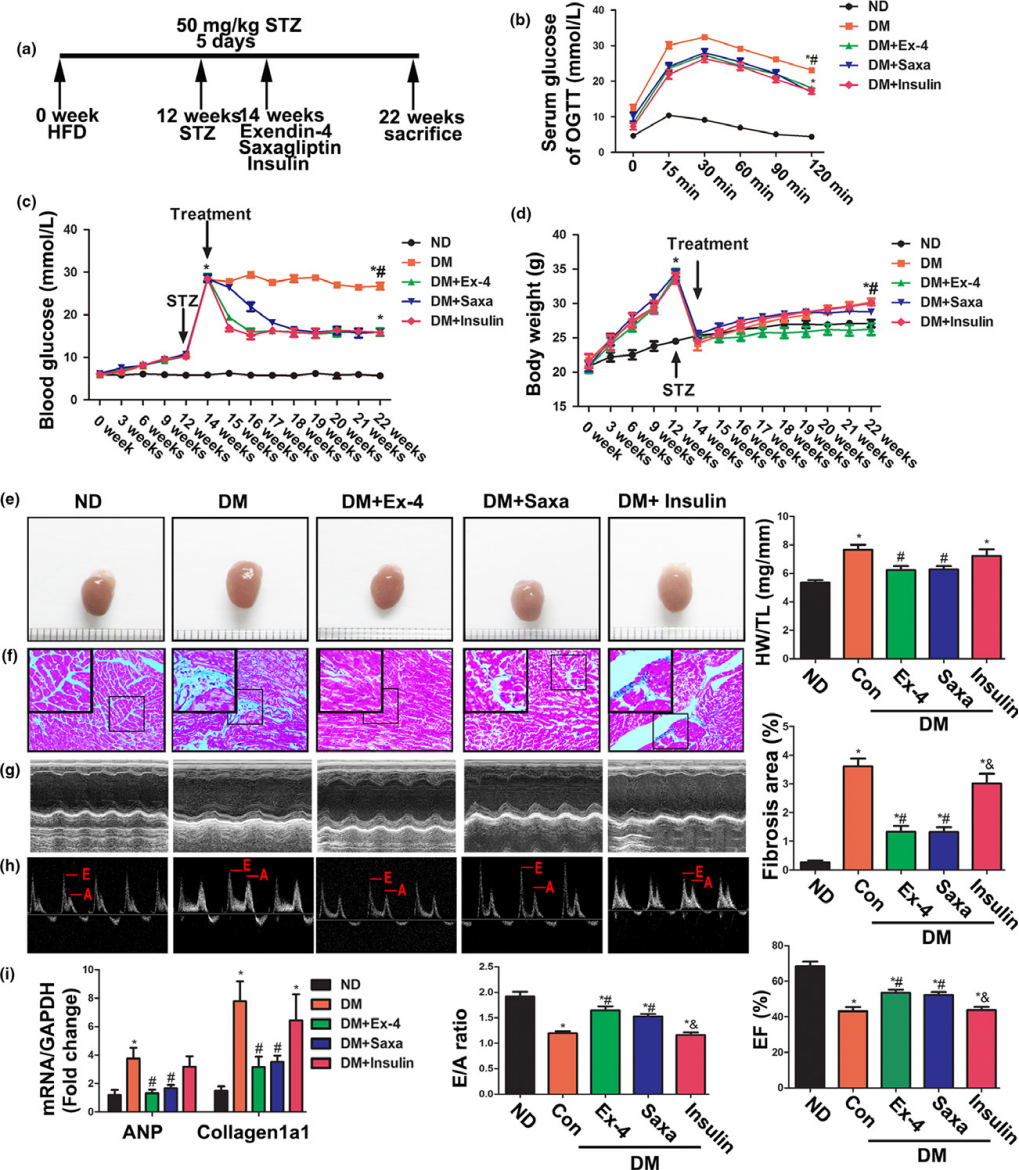
Exendin‐4 and saxagliptin improved metabolic characteristics and cardiac dysfunction in diabetic mice induced by high‐fat diet and low‐dose STZ injection. (a) Schematic of animal experimentation in vivo. Briefly, mice were fed with 60% high‐fat diet (HFD) for 12 weeks, followed by 50 mg/kg STZ treatment for 5 days. At 14 week, experimental diabetes mice divided into different groups were treated with exendin‐4, saxagliptin, or insulin, respectively, for 8 weeks. (b) After an 8‐hr fast, serial tail blood glucose was measured before and after glucose administration (1 g/kg, intraperitoneal injection). *p < .05 vs. ND, ^#^ *p* < .05 DM vs. DM + treatments (exendin‐4, saxagliptin or insulin). *N* = 7–8. (c) Random blood glucose was monitored at different time point. *p < .05 vs. ND, ^#^ *p* < .05 DM vs. DM + treatments (exendin‐4, saxagliptin, or insulin). *N* = 7–8. (d) Body weight was monitored at different time point. *p < .05 vs. ND, ^#^ *p* < .05 DM vs. DM + exendin‐4. *N* = 7–8. (e) Representative images of mice hearts (left) and the ratio of heart weight to tibia length (HW/TL) (right). *p < .05 vs. ND, ^#^ *p* < .05 vs. DM. *N* = 7–8. (f) Representative images (left) and quantitative analysis (right) of fibrosis area stained with Masson's trichrome. *p < .05 vs. ND, ^#^ *p* < .05 vs. DM. *N* = 7–8. (g,h) Echocardiographic data of mice. Ejection fraction (EF) (g), E/A ratio (h). *p < .05 vs. ND, ^#^ *p* < .05 vs. DM, ^&^ *p* < .05 vs. DM + exendin‐4 or saxagliptin. *N* = 7–8. (i) Expression of ANP and collagen 1a1 detected by RT‐PCR. *N* = 6. *p < .05 vs. ND, ^#^ *p* < .05 vs. DM

Type 2 diabetes was induced in mice with low‐dose STZ injections and continuous HFD, exhibiting hyperglycemia, body weight gain, and glucose intolerance compared with normal diet (ND) group (Figure 1a–d). Administration of the GLP‐1R analog Ex‐4 and the DPP‐4 inhibitor saxagliptin for 8 weeks significantly decreased plasma glucose levels and improved glucose tolerance compared with the diabetic mice (DM) group (Figure 1b,c). Saxagliptin provided slower glycemic control than Ex‐4 and insulin but all three groups eventually reached similar glucose levels. Ex‐4 reduced body weight compared with the untreated DM group, but saxagliptin did not (Figure 1d). Both hyperglycemia and dyslipidemia impair heart function in diabetes. To exclude the hypoglycemic capacity when we assessed the cardioprotective effects of Ex‐4 and saxagliptin, we used a 1.5 U/day insulin treatment as a control, as previously reported (Wang et al., 2013). As expected, insulin treatment had the same effects on blood glucose, glucose tolerance, and body weight as exendin‐4 and saxagliptin in DM (Figure 1b–d).

### 2.2 Exendin‐4 and saxagliptin treatments attenuated cardiac remodeling and improved cardiac function in diabetic mice

As shown in Figure 1e, the hearts in the DM were significantly larger than those in the ND mice. In addition, the heart weight to tibial length (HW/TL) ratios (Figure 1e) and atrial natriuretic polypeptide expression (Figure 1i) were higher in the DM than the ND mice. Masson's trichrome stain displayed higher fibrotic areas (Figure 1f), accompanying increases in collagen 1a1 expression (Figure 1i) in the DM than the ND mice. Eight weeks of Ex‐4 and saxagliptin treatments significantly suppressed cardiac fibrosis and hypertrophy in the DM. Hemodynamic and echocardiographic data are shown in Figure 1g,h and Table S1. Relative to the ND mice, the DM were characterized by significant decreases in E/A ratio, ejection fraction (EF), fractional shortening (FS), maximal slope of systolic pressure increment (d*P*/d*t*_max_), minimal slope of diastolic pressure decrement (d*P*/d*t*_min_), and an increase in the left ventricular posterior wall thickness at diastole (LVIDd). All the aforementioned parameters were improved after the Ex‐4 and saxagliptin treatments. However, insulin failed to produce therapeutic effects on diabetic myocardial remodeling and dysfunction (Figure 1e–i). Therefore, the cardioprotection conferred by GLP‐1 may not depend on the ability of this agent to induce hypoglycemia. Taken together, these data showed that it was the activation of the GLP‐1 receptor with Ex‐4 or the elevation of endogenous GLP‐1 with saxagliptin rather than insulin that alleviated cardiac injury in DM.

### 2.3 Exendin‐4 and saxagliptin significantly reduced myocardial lipid accumulation, oxidative stress, inflammation, and apoptosis in vivo

**Figure 2:**
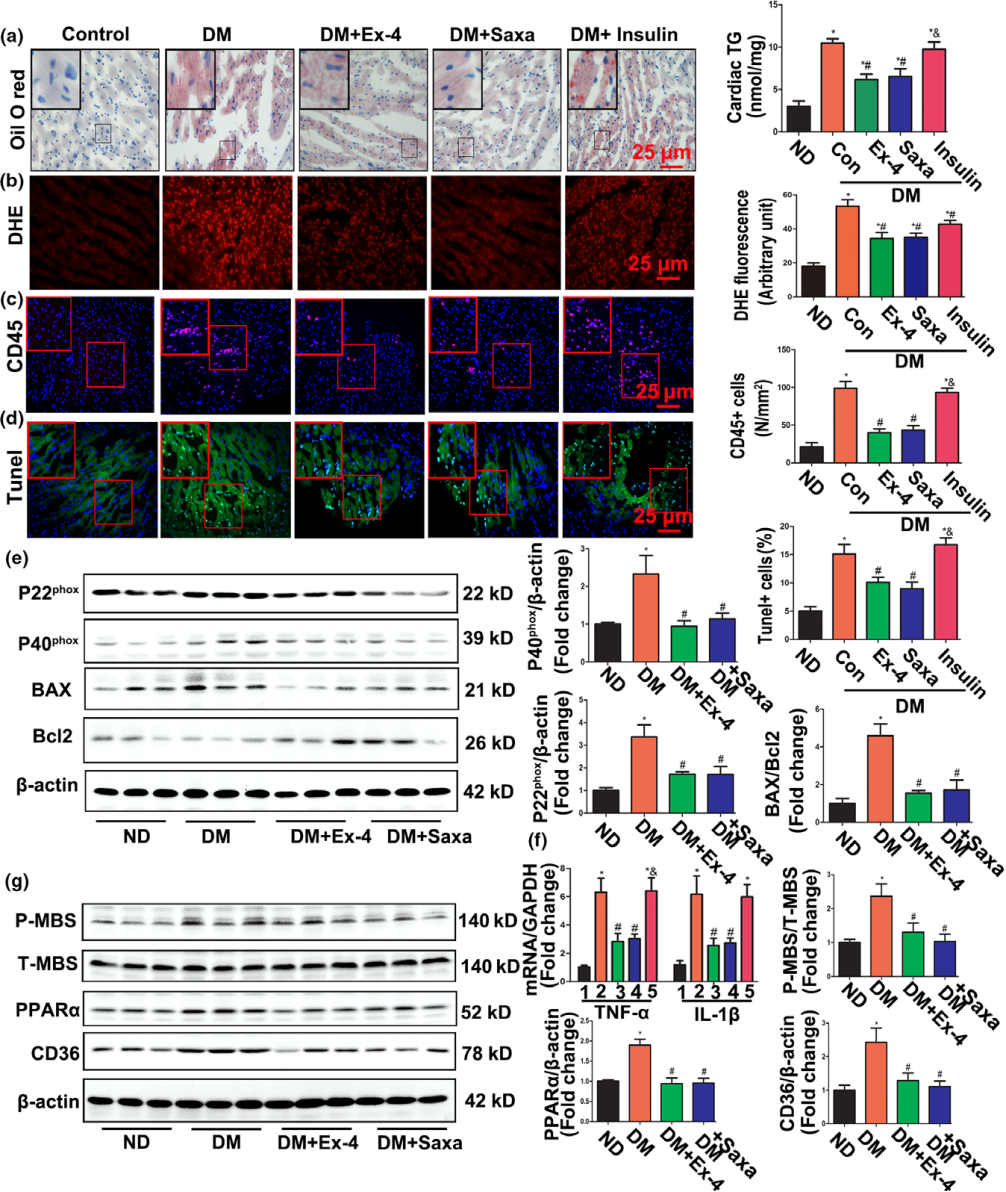
Exendin‐4 and saxagliptin attenuated myocardial lipid accumulation, oxidative stress, inflammation, and apoptosis in diabetic mice. (a) Frozen sections of diabetic hearts were stained with oil red O (left) and the triglycerides (TG) concentrations in hearts tissue were determined (right). *N* = 7–8. Bar = 25 μm. (b) Representative images and quantification of DHE staining of cardiac ROS production. *N *= 7–8. Bar = 25 μm. (c) Inflammatory cells infiltrating in myocardium were stained with CD45 antibody. *N* = 6–8. Bar = 25 μm. (d) Apoptosis index in diabetic hearts was detected by TUNEL assay. *N* = 6–8. Bar = 25 μm. (e) Western blot detects expression of P22^phox^, P40^phox^, Bax, and Bcl2. *N *= three independent experiments. (f) mRNA levels of TNF‐α and IL‐1β detected by RT‐PCR. *N* = 6. *p < .05 vs. ND, ^#^ *p* < .05 vs. DM, ^&^ *p* < .05 vs. DM + Ex‐4 or saxagliptin. (g) Western blot assay for expression of P‐MBS, Total‐MBS, PPARα, and CD36 in diabetic and treated hearts. *p < .05 vs. ND group, ^#^ *p* < .05 vs. DM group

We investigated the effects of Ex‐4 and saxagliptin on the regulation of myocardial lipid metabolism. As shown in Figure 2a, oil red o staining indicated a substantial accumulation of neutral lipid in the hearts of the DM relative to the controls. This effect was reversed by exendin‐4 or saxagliptin but not by insulin. The myocardial triglyceride (TG) levels in heart lysate determined by colorimetric assay were consistent with these results.

Lipotoxicity is associated with increased oxidative stress, inflammation, and apoptosis in DCM (Robertson, Harmon, Tran & Poitout, 2004). DM showed significantly elevated ROS generation and CD45 inflammatory cell infiltration in their myocardia (Figure 2b,c) along with increased expression of the NADPH oxidases P22^phox^ and P40^phox^ and the inflammatory cytokines TNF‐α and IL‐1β (Figure 2e,f). In addition, the apoptosis index (determined by TUNEL staining) and the Bax/Bcl2 ratio (detected by Western blot) were significantly higher in DM hearts than in those of the controls (Figure 2d,e). All these abnormal changes were reversed by an 8‐week treatment with Ex‐4 or saxagliptin. Blood glucose management by insulin also inhibited diabetes‐induced ROS overproduction. Nevertheless, it failed to rectify inflammation or apoptosis. Therefore, Ex‐4 or saxagliptin treatment in DM was cytoprotective, antioxidant, anti‐inflammatory, and anti‐apoptotic.

### 2.4 GLP‐1 receptor activation with exendin‐4 significantly reduced cardiomyocyte lipid accumulation, oxidative stress, and apoptosis in vitro

To confirm the direct protective effects of GLP‐1 on diabetic CMs, H9C2 cardiac myocytes treated with palmitic acid (PA) were used to corroborate the live animal experiments. As shown in Figure S1A, BODIPY staining indicated that Ex‐4 inhibited neutral lipid accumulation in cardiac myocytes. DCFH‐DA and DHE staining verified that Ex‐4 decreased ROS generation induced by PA treatment in cultured CMs (Figure S1B–C). Ex‐4 also inhibited apoptosis and lowered the Bax/Bcl2 ratio in PA‐induced CMs (Figure S1D–E).

### 2.5 GLP‐1 suppressed the diabetes‐related activation of Rho kinase and PPARα in vivo and in vitro

The Rho/Rho‐associated kinase (ROCK) pathway may play important roles in oxidative stress and apoptosis and could be associated with complications of diabetes (Liu, Tan, Lai, Li & Wang, 2016; Zhou & Li, 2012). We examined the effects of GLP‐1 on ROCK activation. As our previous study demonstrated, diabetes mellitus or PA exposure in H9C2 cells significantly elevated phosphorylation of the myosin‐binding subunit (p‐MBS) (Figures 2g and S2A) downstream from ROCK. Treatment with Ex‐4 or saxagliptin lowered the p‐MBS/t‐MBS protein ratio (Figure 2g).

Peroxisome proliferator‐activated receptor alpha (PPARα) is a key regulator of cardiac lipid metabolism. It alone drives the pathologic changes and functional abnormalities in diabetic hearts (Finck et al., 2002, 2003). Therefore, the effects of GLP‐1 on PPARα expression in diabetes were explored. The results showed that GLP‐1 treatment abolished increases in PPARα expression in diabetic hearts and in PA‐treated H9C2 cells (Figures 2g and S2B). Enhancement of the binding of PPARα to PPAR response elements (PPRE) in CMs treated with PA was suppressed by the addition of Ex‐4 (Figure S2C). Furthermore, the increase in the long‐chain FA transporter CD36, a key target gene of PPARα for cardiac palmitate uptake, was significantly attenuated with Ex‐4 and saxagliptin (Figures 2g and S2B).

### 2.6 Exendin‐4 regulated PPARα activation via a PKA/ROCK‐dependent pathway

Previous studies have reported that in diabetes, Ex‐4 suppressed downstream from Rho via a cAMP/protein kinase A (PKA)‐mediated pathway (Wang et al., 2013). We established that Ex‐4 inhibited diabetes‐related activation of Rho kinase and PPARα in vivo and in vitro. Nevertheless, the relationship between the PKA/ROCK‐ and PPARα pathways still remains to be determined. Incubation of cells with the PKA selective inhibitor H89 abrogated the Ex‐4‐induced suppression of PPARα expression, the binding activity of the PPAR response elements (PPRE) primer, and nuclear translocation (Figure S2D–F). All these effects were completely reversed when the ROCK inhibitor fasudil was added (Figure S2D–F). These results suggest that Ex‐4 suppresses diabetes‐induced PPARα activation via a PKA/ROCK‐dependent mechanism.

### 2.7 Exendin‐4 reduced cardiomyocyte lipid accumulation, oxidative stress, and apoptosis via a PPARα‐mediated mechanism in vitro

We then investigated the role of PPARα in GLP‐1‐mediated protection against cardiac lipotoxicity in vitro. As shown in Figure S3A, the Ex‐4‐mediated inhibition of lipid accumulation in cultured H9C2 CMs was completely eliminated by treatment with the PPARα‐selective agonist wy‐14643. Pretreatment with wy‐14643 also abolished the Ex‐4‐mediated suppression of ROS generation induced by PA (Figure S3B–D). Moreover, wy‐14643 pretreatment increased the Bax/Bcl2 ratio and the percentage of AnnexinV/PI‐labeled apoptotic cells relative to those in the Ex‐4 group (Figure S3E–F). Therefore, Ex‐4 reduced lipid accumulation, oxidative stress, and apoptosis in PA‐treated CMs via a PPARα‐mediated mechanism.

### 2.8 Salutary effects of Exendin‐4 on lipotoxicity in isolated adult mouse cardiomyocytes were mediated by the PKA‐ROCK‐PPARα pathway

**Figure 3:**
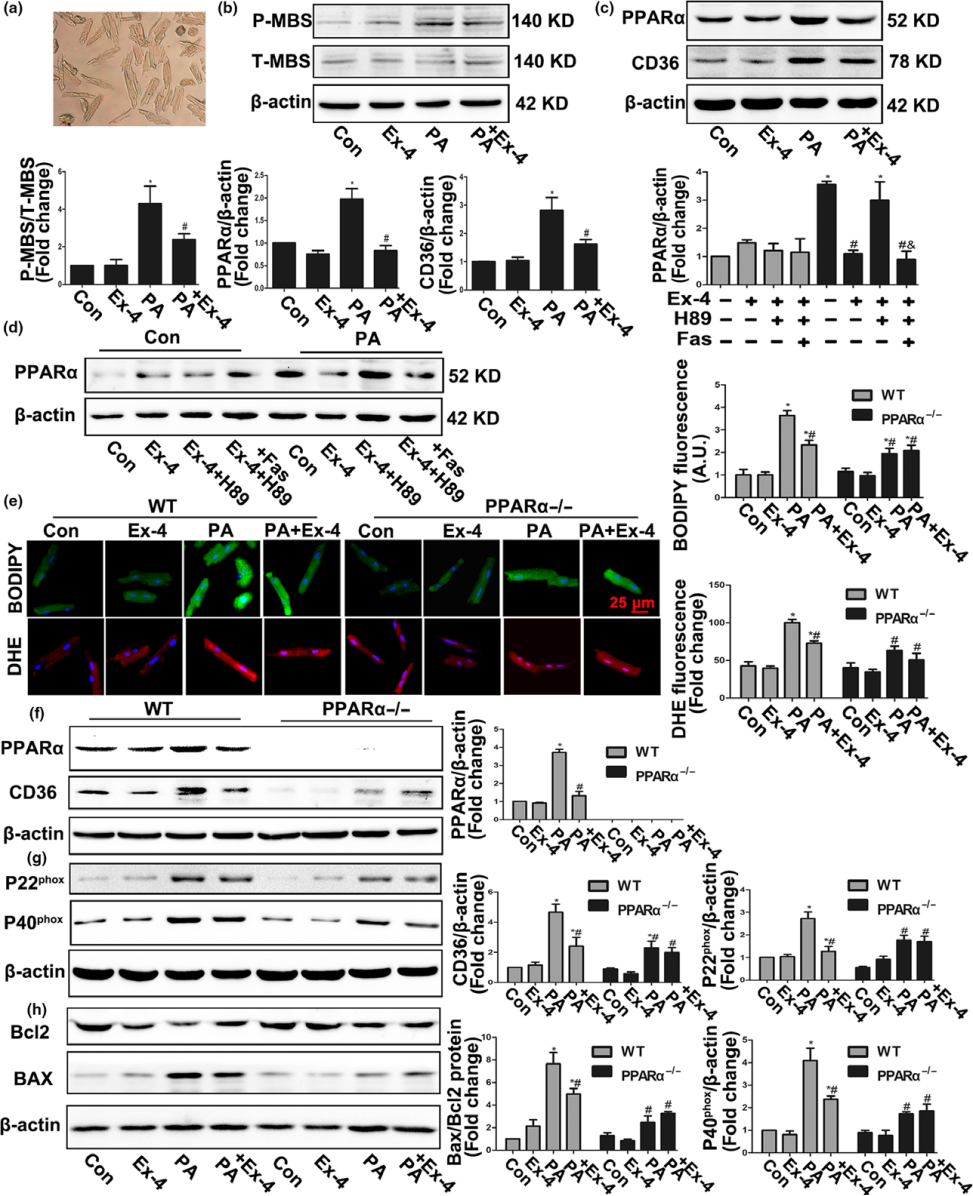
Exendin‐4 attenuated PA‐induced lipid accumulation, oxidative stress, and apoptosis via its control actions on Rock/PPARα pathway in isolated adult mice cardiomyocytes. (a) Adult mice cardiomyocytes were isolated using a Langendorff perfusion system. (b,c) Western blot assay for ROCK (b) and PPARα pathways (c) in PA‐induced WT CMs with or without Ex‐4 treatment. *p < .05 vs. Con, ^#^ *p* < .05 vs. PA group. (d) Expression of PPARα in CMs treated with PA, Ex‐4, H89, and Fasudil. *N* = three independent experiments. *p < .05 vs. Con, ^#^ *p* < .05 vs. PA group, ^&^ *p* < .05 vs. PA + Ex‐4 + H89 group. (e) Representative images and quantification of intracellular ROS production detected by DHE fluorescence and neutral lipids accumulation traced by BODIBY 493/503 after treatment with PA, Ex‐4 in WT, and PPARα KO CMs. *N* = 7. *p < .05 vs. WT Con, ^#^ *p *< .05 vs. WT PA group. (f–h) Expression of PPARα, CD36, P20^phox^, P40^phox^, BAX, and Bcl2 protein detected by Western blot in PA‐induced CMs with or without Ex‐4 treatment. *N* = three independent experiments. *p < .05 vs. WT Con, ^#^ *p* < .05 vs. WT PA group

Considering the metabolic differences between adult and embryonic CMs, we used isolated adult mice CMs (Figure 3a) to validate our conclusions. Consistent with the aforementioned results for H9C2 CMs, Ex‐4 suppressed Rho kinase activity and upregulated the PA‐induced PPARα‐CD36 pathway (Figure 3b,c). We linked the inhibitory effect of Ex‐4 on PPARα with the PKA‐ROCK axis (Figure 3d). Ex‐4 attenuated oxidative stress and apoptosis in adult mouse CMs by reducing excess lipid accumulation (Figure 3e–h). PPARα gene knockout (KO) significantly inhibited lipid accumulation and reduced both oxidative stress and apoptosis (Figure 3e–h). The salutary effects of Ex‐4 were absent in PPARα KO CMs (Figure 3e–h). Therefore, PPARα is a key Ex‐4 target.

### 2.9 PPARα played a central role in the protection against diabetic cardiomyopathy mediated by Ex‐4 in vivo

**Figure 4:**
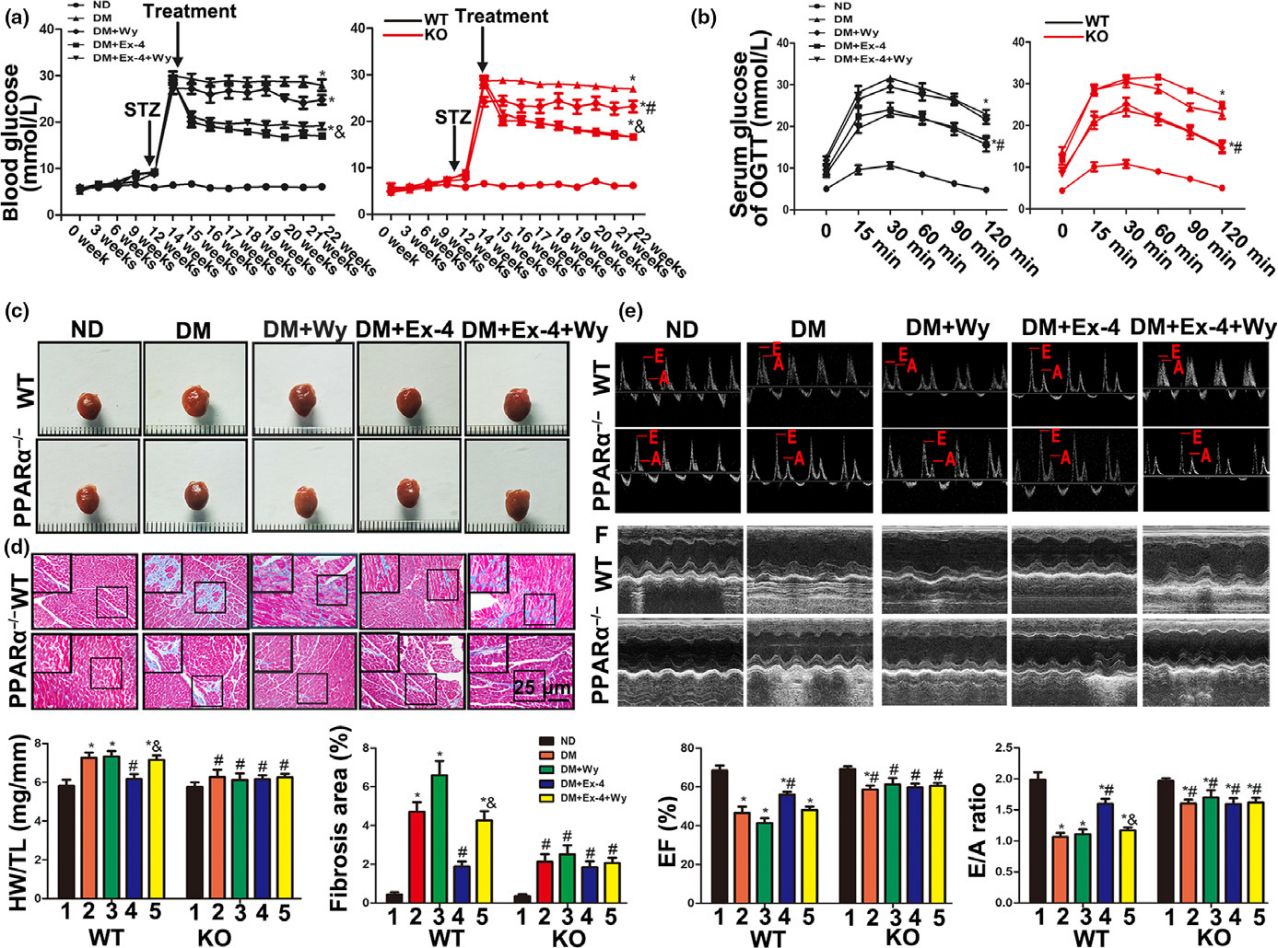
Exendin‐4 reversed cardiac hypertrophy, fibrosis, and dysfunction in diabetic mice induced by high‐fat diet fat and low‐dose STZ injection via PPARα‐mediated pathway. (a) WT and PPARα^−/−^ mice were induced into diabetes by high‐fat diet and low‐dose STZ injection and then treated with placebo, wy‐14643, Ex‐4, or Ex‐4 + wy‐14643, respectively. Random blood glucose was monitored at different time point. *p < .05 vs. WT ND and KO ND, ^#^ *p* < .05 vs. KO DM. ^&^ *p* < .05 vs. WT DM and KO DM. *N* = 6–8. (b) After an 8‐hr fast, serial tail blood glucose was measured before and after glucose administration (1 g/kg, intraperitoneal injection). *p < .05 vs. WT ND and KO ND, ^#^ *p* < .05 vs. KO DM. *N* = 6–8. (c) Representative images of mice hearts (left) and the ratio of heart weight to tibia length (HW/TL) (right). *p < .05 vs. WT ND, ^#^ *p* < .05 vs. WT DM, ^&^ *p* < .05 vs. WT DM + Ex‐4. *N* = 6–8. (d) Representative images (left) and quantitative analysis (right) of fibrosis area stained with Masson's trichrome. *p < .05 vs. WT ND, ^#^ *p* < .05 vs. WT DM, ^&^ *p *< .05 vs. WT DM + Ex‐4. *N* = 6. (e,f) Echocardiographic data of mice. Ejection fraction (EF) (e), E/A ratio (f). *p < .05 vs. WT ND, ^#^ *p* < .05 vs. WT DM, ^&^ *p* < .05 vs. WT DM + Ex‐4. *N* = 6–8

PPARα‐null mice and wy‐14643 were used to understand the mechanisms of PPARα and Ex‐4 in vivo. The results showed that neither PPARα activation nor its deletion participated in the Ex‐4‐mediated blood glucose regulation in DM (Figure 4a,b). There were no significant differences in body weight between treatment groups (Figure S4). Nonetheless, both PPARα deficiency and Ex4 treatment significantly lowered diabetes‐induced heart size increase, HW/TL, and collagen deposition (Figure 4c,d). The wy‐14643 treatment alone did not exacerbate DCM. Nevertheless, the combination of wy‐14643 and Ex‐4 abolished the protective effect of Ex‐4 against diabetes‐induced myocardial hypertrophy and fibrosis (Figure 4c,d). These symptoms were not any less severe in Ex‐4‐treated KO DM than they were in KO DM. Hemodynamics and echocardiography indicated that in wild‐type (WT) DM, the effects of Ex‐4 alone on E/A, EF, FS, and d*P*/d*t* were the opposite of those observed with the combined Ex‐4/wy‐14643 treatment (Figure 4e,f and Table S2). PPARα deficiency had essentially the same effects on cardiac function as the Ex‐4‐treatment (Figure 4e,f and Table S2). However, there were no improvements in cardiac function in PPARα‐null DM treated with Ex‐4 compared with PPARα KO DM. In summary, exendin‐4 protected against diabetes cardiomyopathy via a PPARα mechanism.

### 2.10 Exendin‐4 reduced myocardial lipid accumulation, oxidative stress, and apoptosis via a PPARα‐mediated mechanism in vivo

**Figure 5:**
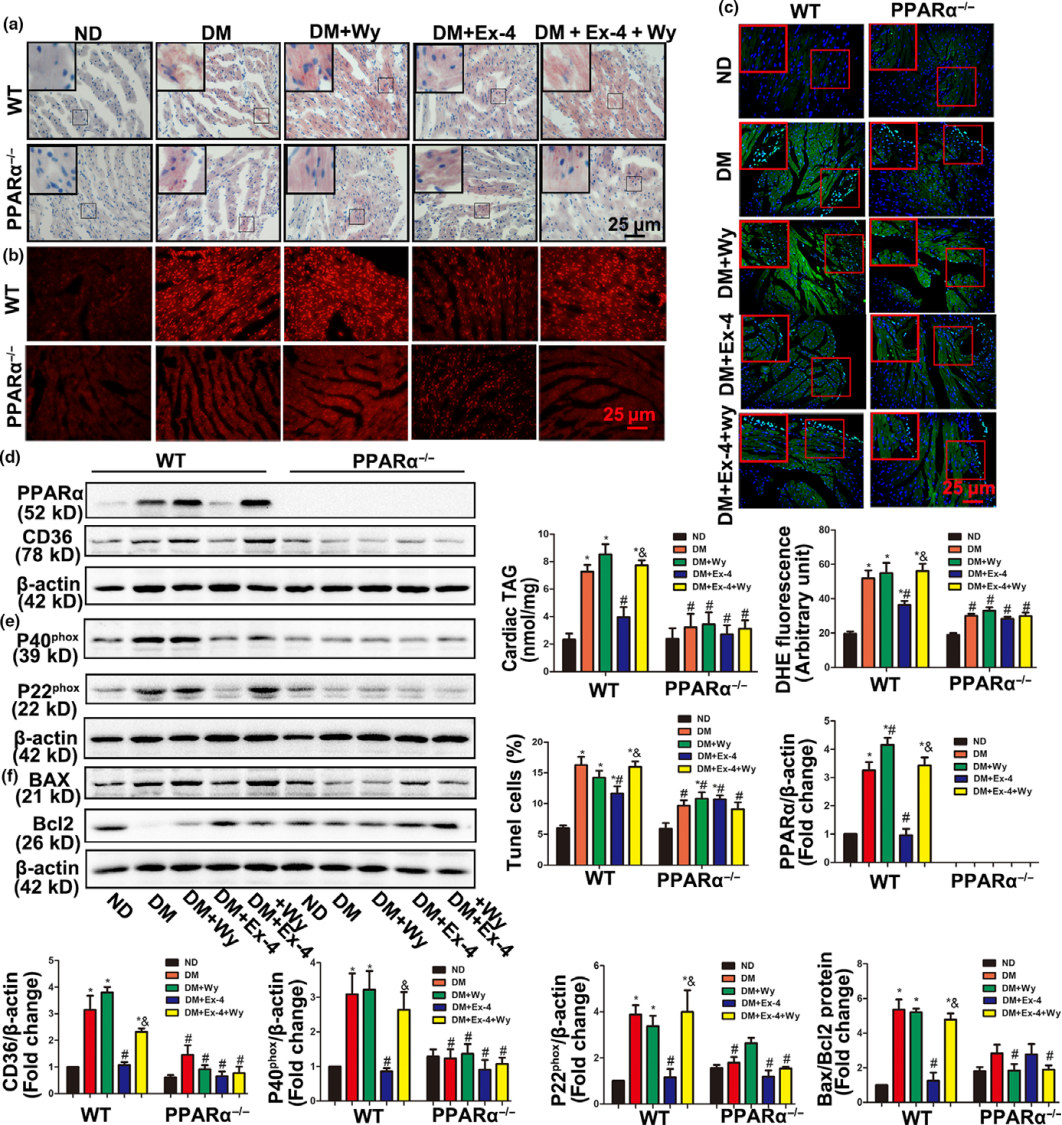
Exendin‐4 attenuated lipid accumulation, oxidative stress, and apoptosis in diabetic mice through PPARα‐mediated mechanism. (a) Frozen sections of diabetic hearts were stained with oil red O and the triglycerides (TG) concentrations in hearts tissue were determined. *N* = 6–8. Bar = 25 μm. (b) Representative images and quantification of DHE staining of cardiac ROS production. *N *= 7. Bar = 25 μm. (c) Apoptosis index in diabetic hearts was detected by TUNEL assay. *N *= 6–8. Bar = 25 μm. (d–f) Expression of PPARα and CD36, P20^phox^ and P40^phox^, Bax and Bcl2 protein were detected by Western blot. *p < .05 vs. WT ND, ^#^ *p* < .05 vs. WT DM, ^&^ *p* < .05 vs. WT DM + Ex‐4

As shown in Figure 5a, the combination of Ex‐4 and wy‐14643 elevated myocardial lipid accumulation more than the Ex‐4 treatment alone in WT DM. PPARα deficiency mimicked the effects of Ex‐4. In addition, Ex‐4 treatment of PPARα KO in DM did not inhibit lipid accumulation relative to the WT DM + Ex‐4 group. Therefore, Ex‐4 attenuated diabetes‐induced lipid metabolic disorder mainly in a PPARα‐dependent manner (Figure 5a). Expression of the long‐chain FA transporter CD36 significantly decreased when the PPARα protein was absent in mice (Figure 5d). Myocardial oxidative stress and apoptosis displayed the same trend upon evaluation by staining and Western blotting (Figure 5b,c,e,f). These data suggest that exendin‐4 decreases myocardial lipid accumulation, oxidative stress, and apoptosis via a PPARα‐mediated mechanism.

### 2.11 Cardiac‐specific PPARα overexpression induced by the adeno‐associated virus serotype‐9 (rAAV‐9) reversed the salutary effects of exendin‐4 on diabetic cardiomyopathy

**Figure 6:**
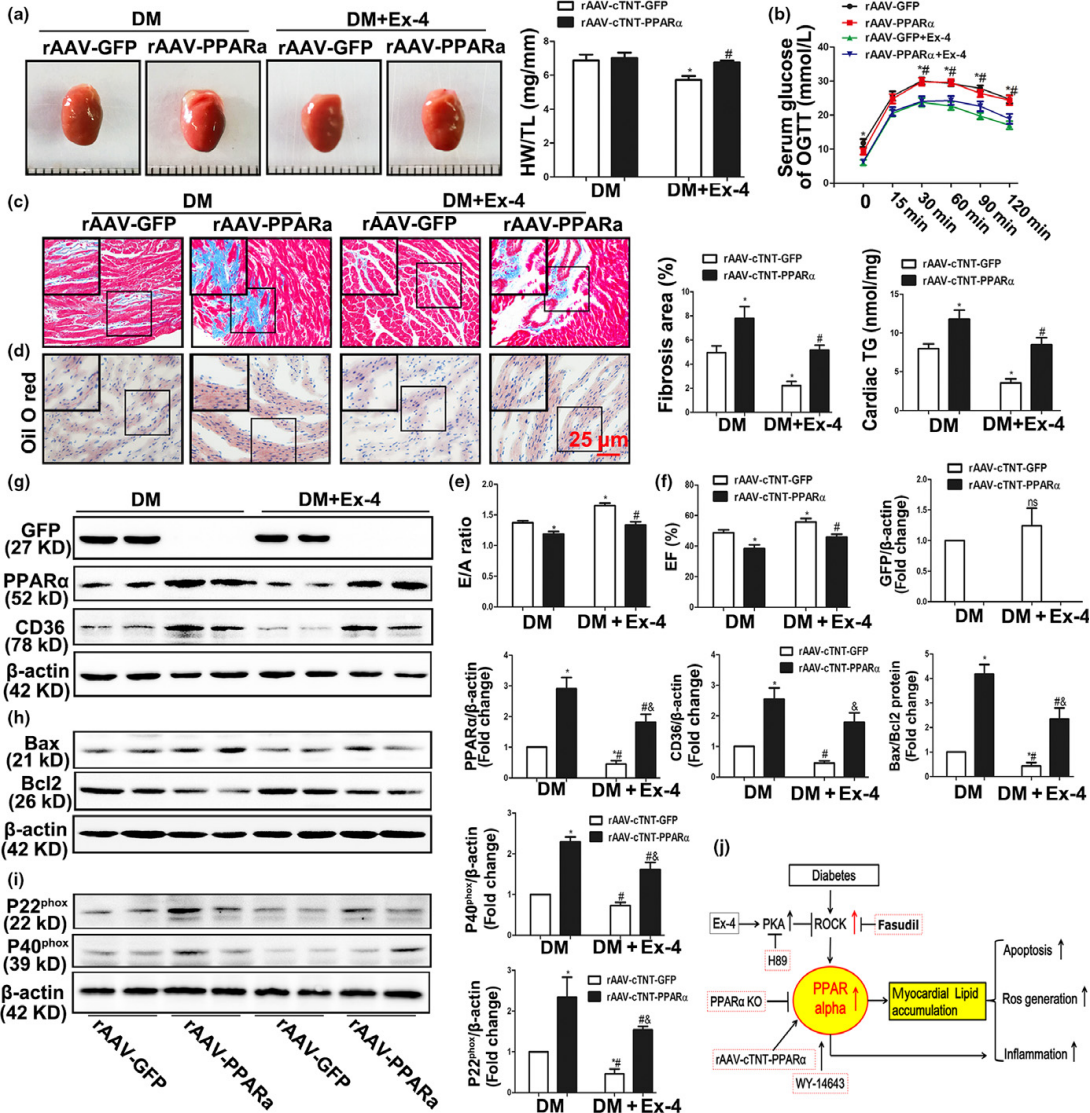
Cardiac‐restricted PPARα overexpression reversed Ex‐4's benefits in diabetic cardiomyopathy. (a) Representative hearts images (left) of rAAV‐GFP and rAAV‐PPARα diabetic mice (DM) with or without Ex‐4 treatment and the ratio of heart weight to tibia length (HW/TL). *p < .05 vs. rAAV‐GFP DM, ^#^ *p* < .05 vs. rAAV‐GFP + Ex‐4 treated mice. *N *= 7–9. (b) OGTT was measured before and after glucose administration (1 g/kg, intraperitoneal injection). *p < .05 vs. rAAV‐GFP + Ex‐4 DM, ^#^ *p* < .05 vs. rAAV‐PPARα + Ex‐4 treated mice. *N *= 7–9. (c) Cardiac fibrosis area was stained with Masson's trichrome. *p < .05 vs. rAAV‐GFP DM, ^#^ *p* < .05 vs. rAAV‐GFP + Ex‐4 treated mice. *N* = 7–9. Bar = 25 μm. (d) Frozen sections of diabetic hearts were stained with oil red O and the triglycerides (TG) concentrations in hearts tissue were determined. *p < .05 vs. rAAV‐GFP DM, ^#^ *p* < .05 vs. rAAV‐GFP + Ex‐4 treated mice. *N *= 7. Bar = 25 μm. (e,f) Echocardiographic data of mice. Ejection fraction (EF) (e), E/A ratio (f). *p < .05 vs. rAAV‐GFP DM, ^#^ *p* < .05 vs. rAAV‐GFP + Ex‐4 treated mice. *N *= 8. (g–i) Expression of GFP, PPARα and CD36, P20^phox^ and P40^phox^, Bax and Bcl2 protein were detected by Western blot. *p < .05 vs. rAAV‐GFP DM, ^#^ *p* < .05 vs. rAAV‐GFP + Ex‐4 treated mice. *N* = three independent experiments. (j) Mechanism diagram of full text

Previous studies reported the beneficial effects of Ex‐4 on cardiac microvascular endothelium and inflammatory cells. Therefore, we induced cardiac‐specific PPARα overexpression in mice via rAAV‐cTNT‐PPARα virus treatment to determine whether our conclusions were based on CMs alone. As expected, the rAAV‐cTNT‐GFP and rAAV‐cTNT‐PPARα viruses significantly increased the expression of the genes they bore in mouse hearts (Figure S5A). Both rAAV‐GFP and rAAV‐PPARα mice developed stable hyperglycemia, glucose intolerance (Figures 6b and S5B). These were corrected by Ex‐4 treatment. However, no difference was observed between the Ex‐4 treatment and control groups in terms of weight gain (Figure S5C). Cardiac‐restricted PPARα overexpression failed to increase heart size and HW/TL relative to those of rAAV‐GFP DM (Figure 6a). Nevertheless, there were substantial increases in myocardial fibrotic area, cardiac lipid accumulation, and triglyceride (TG) content (Figure 6c,d). DM overexpressing PPARα also presented with more severe cardiac damage and poorer diastolic and systolic functions than rAAV‐GFP DM (Figure 6e,f and Table S3). They also showed higher myocardial apoptosis (Figure 6h) and oxidative stress (Figure 6i). Ex‐4 treatment in GFP DM improved heart structure and function (Figure 6a–i) compared with the untreated control groups. This amelioration may be ascribed to the negative effects of Ex‐4 on the PPARα‐CD36 pathway (Figure 6g). However, compared with Ex‐4‐ rAAV‐GFP DM, cardiac‐restricted PPARα overexpression partly counteracted the salutary effects of Ex‐4 on diabetes‐related cardiac dysfunction, myocardial apoptosis, and oxidative stress (Figure 6a–i). Compared with rAAV‐PPARα DM, Ex‐4‐rAAV‐PPARα DM group showed a significant decrease in PPARα/CD36 protein expressions, apoptosis, and oxidative stress. Although there was no statistical difference, improving trends in myocardial fibrotic area, TG content and cardiac function were also observed in Ex‐4‐rAAV‐PPARα DM. These data implied that there are potentially additional effects of Ex‐4 beyond CMs. Taken together, our results indicated that the negative effects of Ex‐4 on the CM PPARα‐CD36 pathway at least partly explained its ability to protect against DCM (Figure 6j).

## 3 DISCUSSION

In the present study, we generated an obese/insulin resistance mouse model mimicking human type 2 diabetes with continuous HFD and low‐dose STZ injection. The treated mice developed severe hyperglycemia, obesity, impaired insulin secretion, and stable insulin resistance. In addition, this model could be easily and rapidly established in KO mice. Here, we showed that both the GLP‐1 analog exendin‐4 and the DPP‐4 inhibitor saxagliptin improved the diabetes phenotype, mitigated heart damage, hyperglycemia, insulin resistance, myocardial remodeling, and cardiac dysfunction. These effects occurred partially independently of the established glucose‐lowering capacities of these agents. Furthermore, exendin‐4 modified the abnormalities in the ROCK and PPARα pathways induced in vitro by PA. For this reason, we investigated the connection between PPARα‐mediated lipid regulation and the cardiac benefits of Ex‐4. The therapeutic effects of Ex‐4 were mimicked by PPARα deficiency but reversed by the PPARα agonist wy‐14643 and by cardiac‐specific PPARα overexpression. These observations corroborated our theory that GLP‐1 ameliorates myocardial lipid uptake and mitigates the resultant lipotoxicity by controlling PPARα.

It has been reported that DPP‐4 inhibitors have more complex and broader effects than GLP‐1. They inhibited the enzymatic degradation of dozens of chemokines and peptide hormones besides native GLP‐1 (Scheen, 2017). Several studies have discussed the differences between the DPP‐4 inhibitors and the GLP‐1 analogs on hypoglycemic action, cardiovascular outcomes, and body weight control (Nauck, Meier, Cavender, Abd El Aziz & Drucker, 2017). Currently, saxagliptin and other DPP‐4 inhibitors are still regarded as important drugs preventing the enzymatic degradation of endogenous GLP‐1. In our study, however, exendin‐4 only moderately controlled weight but had a faster hypoglycemic action than saxagliptin. There were no significant differences between the two agents in terms of their effects on cardiac function, myocardial lipid accumulation, apoptosis, or oxidative stress. It is possible that the benefits of saxagliptin were mainly GLP‐1‐dependent in our diabetic models.

Cardiac cell apoptosis is the most frequently proposed mechanism of DCM progress (Ouyang, You & Xie, 2014). Apoptotic cell death is also regarded as a terminal junction of various molecular mechanisms. It contributes to cardiac remodeling by destroying contractile units and inducing compensatory myocardial cell hypertrophy and reparative fibrosis (Kusminski, Shetty, Orci, Unger & Scherer, 2009). The rate of CM apoptosis in patients with diabetes is 85‐fold greater than that in nondiabetics (Ho, Liu, Liau, Huang & Lin‐Shiau, 2000). Diabetes‐induced CM apoptosis has been associated with excessive generation of reactive free radicals even though other inductive pathways exist as well (Dorn, 2009; Robertson et al., 2004). Increased ROS production and reduced antioxidant levels in diabetes have been widely documented in previous reports (Fiordaliso et al., 2004; Houstis et al., 2006). Earlier studies showed that CMs incubated with GLP‐1 or its analogs remained viable and lowered ROS levels and apoptosis rates in both diabetic and nondiabetic models (Inoue et al., 2015; Raab, Vuguin, Stoffers & Simmons, 2009; XiaoTian et al., 2016). Nevertheless, these reports failed to address the possible mechanisms responsible for these effects. In the present study, we observed decreases in CM apoptosis and oxidative stress in the presence of exendin‐4 or saxagliptin and elucidated their modes of action.

Some studies have attributed the benefits of Ex‐4 on DCM to its effects on infiltrating macrophages, cardiac microvascular injury, and mitochondrial dysfunction (Tate et al., 2016; Wang et al., 2013; Wassef et al., 2017). However, the present study mainly focused on the effects of GLP‐1 on lipid regulation because, along with hyperglycemia, lipid accumulation and toxicity play key roles in DCM (Kusminski et al., 2009; Yang et al., 2007). The lack of glycemic control in cardiovascular disease progress in obese and T2DM patients underscores the importance of lowering cardiac steatosis in them. Excessive epicardial fat accumulation closely linked to cardiometabolic disruptions and mortality in T2DM patients through secretion of lipids, adipokines, and pro inflammatory and oxidative factors (Fitzgibbons & Czech, 2014; Gonzalez, Moreno‐Villegas, Gonzalez‐Bris, Egido & Lorenzo, 2017). Unoxidized FA accumulation in cardiac myocytes impairs energy metabolism and aggravates mitochondrial dysfunction, ROS overproduction, and lipoapoptosis (Drosatos & Schulze, 2013; Rodrigues et al., 1995). Several recent studies concluded that strategies to minimize ectopic fat accumulation and lipotoxicity have direct cardioprotective effects (Mori et al., 2014; Yang et al., 2007). To the best of our knowledge, the present study is the first to revealed that both exendin‐4 and saxagliptin significantly reduced lipid content in CMs both in vivo and in vitro by controlling the PPARa‐CD36 pathway, which is a major regulatory signal in cardiac FA metabolism. The long‐chain FA transporter CD36 is responsible for >60% of the cardiac FA uptake (Angin et al., 2012). Heart‐specific CD36 deficiency prevents myocardial lipid accumulation and rescues cardiac dysfunction. Therefore, CD36 may be a key therapeutic target for DCM (Yang et al., 2007). In the present study, we report for the first time that the restricted expression of myocardial CD36 was associated with the cardiac benefits of exendin‐4 treatment.

PPARα had been widely accepted as a transcriptional switch for various genes involved in cardiac FA uptake and oxidation. PPARα may hasten the progress of DCM (Finck et al., 2002, 2003). In the present study, PPARα KO mice failed to develop DCM. Cardiac‐specific PPARα overexpression showed more severe DCM. WT DM mice receiving wy‐14643 treatment alone presented with higher myocardial lipid levels and fibrosis severity than WT DM. Nevertheless, no significant differences were observed between the two groups in terms of cardiac function, oxidative stress, or apoptosis. It is possible that wy‐14643 alone does not induce cardiac damage as severe as that caused by cardiac‐specific PPARα overexpression. On the other hand, wy‐14643 could reverse the PPARα inhibition promoted by exendin‐4. The present study demonstrates that exendin‐4 suppresses the PPARα expression and nuclear translocation induced by diabetes mellitus. These are the key mechanisms explaining the lipid‐lowering property and cardioprotective effect of Ex‐4. In contrast, contradictory conclusions about the role of PPARα on DM have also been documented (Baraka & AbdelGawad, 2010; Young et al., 2001). CMs chronically exposed to FA showed relatively lower PPARα expression and treated with the PPAR agonist fenofibrate showed suppression of PA‐induced apoptosis (Young et al., 2001). We propose that the model and treatment style differences among these studies account for these discrepancies.

Our data indicate that Ex‐4 inhibited the ROCK/PPARα/CD36 pathway by PKA activation. The RhoA/ROCK pathway is a key mediator of oxidative stress‐mediated cell injury (Liu et al., 2016; Zhou & Li, 2012). Our previous study demonstrated that the RhoA/Rock pathway is strongly activated in patients with diabetes (Liu et al., 2016). Moreover, there is evidence that the RhoA/ROCK pathway contributes to DM pathogenesis both in vitro and in vivo (Furukawa et al., 2005; Wang et al., 2013). GLP‐1 may attenuate the oxidative stress induced in cardiac microvascular endothelial cells by high glucose via the activation of cAMP/PKA and the inhibition of downstream ROCK activity (Wang et al., 2013). However, these results do not explain the beneficial effects of exendin‐4 on cardiac lipotoxicity. In the current study, our data clearly linked the GLP‐1/PKA/ROCK regulatory axis and the PPARα/CD36 lipid metabolic signal.

In conclusion, we demonstrate that the GLP‐1 analog exendin‐4 improved the structural and functional abnormalities of diabetic hearts at least in part by inhibiting the PPARα‐mediated lipid accumulation and toxicity regulated by the PKA/ROCK pathway. GLP‐1 analogs may be useful as therapies for lipotoxic cardiomyopathy as well as hyperglycemia.

## 4 EXPERIMENTAL PROCEDURES

### 4.1 Animal model and experimental design

The animal experiments conformed to the Guide for the Care and Use of Laboratory Animals published by the United States National Institutes of Health (NIH Publication No. 85‐23, revised 1985). All experimental protocols were approved by the Experimental Animal Research Committee of Tongji Medical College, Huazhong University of Science and Technology, Wuhan, China.

#### 4.1.1 Experiment 1

Male C57BL/6 mice (16–20 g body weight) were purchased from the Experimental Animal Center of Wuhan University (Wuhan, China). After acclimatization for 1 week, the mice were initially administered either a normal chow diet (ND) or 60% HFD (diet #D12492, Research Diets, Inc., New Brunswick, NJ) for 12 weeks. The HFD mice were then intraperitoneally injected with 50 mg/kg body weight STZ (Sigma‐Aldrich Corp., St. Louis, MO, USA). The ND mice received equivalent volumes of 0.1 m citrate buffer for 5 days. Serum glucose levels were measured by tail blood glucometry (Bayer Corp., Mishawaka, IN, USA) 2 weeks after the first injection. Mice with random blood glucose levels >16.7 mm were considered diabetic and were recruited for the subsequent experiments (Finck et al., 2002). The DM were randomly divided into four groups: (i) DM; (ii) DM subcutaneously injected with exendin‐4 (Ex‐4) at 100 μg kg^−1^ day^−1^; (iii) DM orally treated with saxagliptin (Saxa) at 10 mg kg^−1^ day^−1^; (iv) DM treated with 1.5 U insulin.

#### 4.1.2 Experiment 2

The PPARα KO on a C57Bl/6J background was purchased from Jackson Labs (Bar Harbor, ME, USA). Purebred wild‐type littermate mice were used. Diabetes was induced as described in Experiment 1. PPARα^−/−^ (KO) mice were randomly assigned to receive the following treatments: (i) control group with ND; (ii) DM treated with PBS for 8 weeks; (iii) DM treated with wy‐14643 (100 μm in drinking water); (iv) DM treated with Ex‐4 (100 μg kg^−1^ day^−1^); and (5) DM treated with wy‐14643 (100 μm in drinking water) plus Ex‐4 (100 μg kg^−1 ^day^−1^). WT mice were also used as controls for each group.

#### 4.1.3 Experiment 3

The rAAVs (type 9) bearing cardiac troponin T (cTNT) promoter and expressing GFP and PPARα proteins were prepared by triple plasmid cotransfection in HEK293T cells as previously described (Li et al., 2016). Detailed information is provided in the online‐only Data Supplement entitled Expanded Methods. A single tail vein injection of the rAAV‐cTNT‐GFP or the rAAV9‐cTNT‐PPARα (2 × 10^11^ vector genomes (vg) per mouse) was performed in adult male C57BL/6 mice (8–10 weeks). Two weeks after injection, the animals were developed into diabetic models according to the method described in Experiment 1. Cardiac‐specific GFP‐ or PPARα‐overexpressing mice were randomly assigned to four groups: (i) rAAV‐GFP DM; (ii) rAAV‐GFP DM treated with Ex‐4 (100 μg kg^−1^ day^−1^); (iii) rAAV9‐PPARα DM; and (iv) rAAV9‐PPARα DM treated with Ex‐4 (100 μg kg^−1^ day^−1^).

### 4.2 Statistical analysis

All data are presented as means ± standard error of the means unless otherwise stated. Blood glucose and body weight at the 22nd week, OGTT at 120 min, heart weight:tibia length, fibrotic area, cardiac function parameters, Western blot densitometry, real‐time polymerase chain reaction data, and fluorescence intensity in the first animal and most cell experiments were analyzed by one‐way ANOVA. The Student–Newman–Keuls post hoc test was used to evaluate differences between groups. Data from the second and third animal experiments and from the adult mouse CM experiments shown in Figure S3E–H were analyzed by two‐way ANOVA. The Tukey's post hoc test was used to evaluate differences between groups. *p *<* *.05 was considered statistically significant. All statistical tests were performed using GraphPad Prism v. 5.0 (GraphPad Software, San Diego, CA, USA) and SPSS v. 18.0 (IBM Corp., Armonk, NY, USA).

Other details of the experimental procedures are available in the Supporting information.

## CONFLICT OF INTEREST

The authors declare no potential conflict of interests relevant to this article.

## AUTHORS' CONTRIBUTION

Lujin Wu conceived and performed the experiments, collected and analyzed data, and wrote the manuscript. Ke Wang and Wei Wang conducted parts of the animal experiments and collected the data. Zheng Wen and Peihua Wang conceived the experiments and reviewed the manuscript. Lei Liu and Dao Wen Wang conceived and designed the experiments and reviewed and edited the manuscript.
