# Supplementary material for: Glucagon‐like peptide‐1 ameliorates cardiac lipotoxicity in diabetic cardiomyopathy via the PPARα pathway
Source: Aging Cell. 2018 Apr 16;17(4):e12763. doi: 10.1111/acel.12763 (PMC6052396; doi:10.1111/acel.12763)
Supplement: Supplementary file 1 [file ACEL-17-na-s001.doc]

**Glucagon-like peptide-1 ameliorates cardiac lipotoxicity in diabetic cardiomyopathy via the PPARα pathway**

Lujin Wu^1,2^, Ke Wang^3^, Wei Wang^1,2^, Zheng Wen^1,2^, Peihua Wang^1,2^, Lei Liu^1,2*^, Dao Wen Wang^1,2*^

^1^ Division of Cardiology, Department of Internal Medicine, Tongji Hospital, Tongji Medical College, Huazhong University of Science and Technology, Wuhan 430030, China

^2^ Hubei Key Laboratory of Genetics and Molecular Mechanism of Cardiologic Disorders, Wuhan 430030, China

^3^ Department of Neonatal Medicine, The Central Hospital of Wuhan, Tongji Medical College, Huazhong University of Science and Technology, Wuhan 430030, China

*Corresponding author: Dao Wen Wang, M. D., Ph. D.; or Lei Liu, M. D., Ph. D.

Division of Cardiology, Department of Internal Medicine, Tongji Hospital, Tongji Medical College, Huazhong University of Science & Technology, 1095# Jiefang Ave, Wuhan 430030, China

Tel. & Fax: 86-27-8366-3280

Email: [dwwang@tjh.tjmu.edu.cn](mailto:dwwang@tjh.tjmu.edu.cn ) or [leiliu@tjh.tjmu.edu.cn](mailto:leiliu@tjh.tjmu.edu.cn)

**Running title:** *GLP-1 and cardiac lipotoxicity*

**Key words:** GLP-1; DPP-4; Lipotoxicity cardiomyopathy; Apoptosis; PPARα

**Cell culture and treatment**

Rat embryonic cardiomyoblast-derived H9c2 cells were obtained from the Cell Bank of the Chinese Academy of Sciences (Shanghai, China). The cells were divided into following groups according to the different needs of experiments: (1) Control group (normal glucose concentration, 5.5 mM); (2) Control treated with or without exendin-4, H89, Fasudil or wy-14643; (2) 0.5mM Palmitic acid (PA) group; (3) PA group treated with or without exendin-4, H89, Fasudil or wy-14643.

**Adult mice cardiomyocyte isolation**

Adult mice cardiomyocytes (CMs) were isolated according to our built protocol as previous described . In detail, hearts of Wild-type mice or PPARα KO mice were removed and the coronary arteries perfused via the aorta at 4.0 mL/min (37°C) using a Langendorff system for 5 minutes with a Ca^2+^-free Tyrope’s (CT) solution. The composition of CT (in mM) was: NaCl (120.00), KCl (5.40), HEPES (20.00), NaH_2_PO_4_ (0.52), MgCl_2_ (3.50), taurine (20.00), creatine (10.00), glucose (11.10). The pH was adjusted to 7.35~7.38 with NaOH. Enzymatic digestion was initiated by adding 1.0 mg/mL of type I collagenase (Worthington Biochemical) and 0.1 mg/mL of type XIV protease (Sigma-Aldrich) to the perfusion solution. After approximately 10 minutes, the left ventricle was removed and cut into strips and then mixed to yield a single-cell suspension in CT solution containing 0.7% BSA. The calcium concentration in CT solution was raised in this suspension via stepwise increments until 1.0 mM was reached. Then, Adult CMs were stimulated by PA with or without Ex-4 treatment for western blotting, ROS production and lipids determination.

**Construction of recombinant adeno-associated virus (rAAV) vectors**

The rAAV-9 system was a kind gift from Dr. Xiao Xiao (University of North Carolina at Chapel Hill). Expression plasmids carrying GFP and full-length PPARα sequence was synthesized by BGI Tech (Shenzhen, China) and then cardiac troponin T (cTNT) promoter were inserted. The rAAV vectors bearing the cardiac troponin T (cTNT) promoter driving the expression of enhanced green fluorescent protein (AAV9-cTNT-GFP) and mouse PPARα (AAV9-cTNT-PPARα) were prepared by triple plasmid co-transfection in human embryonic kidney 293 cells and then purified as described previously .

**Oral glucose tolerance test (OGTT) experiment**

After fasted for overnight, blood glucose of experiment mice were analyzed by tail blood glucose monitoring. Then, OGTT was conducted by intraperitoneal injection of 1g/kg body weight glucose (D-glucose, Sigma 25mg/ml in saline) at time 0 as previously described . Blood glucose was detected at 15, 30, 60, 90 and 120 min after glucose treatment.

**Haemodynamic measurements and echocardiography**

At the end of the study, echocardiographic examinations were performed under light (1–2%) isoflurane anaesthesia using a high-resolution imaging system with a 30-MHz high frequency scanhead (Visual SonicsVevo-1100, Visual Sonics Inc., Toronto, Canada) as previously described . Left ventricle haemodynamic measurements were performed under intraperitoneal injection of 90 mg/kg ketamine and 10 mg/kg xylazine using a Millar Catheter System via the left carotid artery as described previously .

**Measurement of Myocardium Fibrosis and inflammatory cells infiltration** Masson’s trichrome staining and CD45 staining were performed in cardiac tissue with paraffin-embedded sections as previously described . The percent of myocardium fibrosis area and CD45 positive cells were quantified by the color image analyzer (Image-Pro Plus; Media Cybernetics, Rockville, MD, USA).

**TUNEL staining for assessment of apoptosis in heart slice**
Apoptotic cell death in the heart was detected in situ by terminal deoxynucleotidyl transferase (TdT)-mediated dUTP-biotin nick end-labeling (TUNEL) staining of fragmented DNA using an In situ Cell Apoptosis Detection Kit (Beyotime, Shanghai, China). The procedure was performed according to the manufacturer’s instructions. Briefly, cells were fixed with 4% paraformaldehyde and permeabilized by 0.3% Triton X-100 and then labeled by incubation (1h, 37°C) with terminal deoxynucleotidyltransferase and nucleotide mixture containing fluorescein isothiocyanate-conjugated dUTP. Cells nuclear were stained by 4’,6-diamidino-2-phenylindole fluorescent dye (DAPI), and the TUNEL positive and total nuclei were observed under a laser scanning confocal microscope (Nikon, Japan).

**Flow cytometry analysis of Apoptosis**

According to our experimental design, we treated H9C2 cells with PA and with or without GLP-1 receptor agonist Exendin-4 and PPARα agonist Wy-14643 and the doses were given as aforementioned. To evaluate cell apoptosis, cells incubated with Annexin V-FITC/PI Apoptosis Detection Kit (Invitrogen) were analyzed with a FACStar Plus flow cytometer (BD, Franklin Lakes, NJ) as previously described .

**Real-Time Polymerase Chain Reaction and Western Blot Analysis**
Total RNA was isolated with Trizol reagent (Invitrogen) according to the manufacturer’s instructions and reverse-transcribed by M-MLV First-Strand cDNA Synthesis Kit (Invitrogen) . The mRNA levels were quantified by qRT-PCR using Power SYBR Green PCR Master Mix (Invitrogen) with the primers listed in Table S3. GAPDH served as an internal control and the results analyzed with the 2^-ΔΔCt^ method.

Protein lysates were extracted using Boster Kit according to the manufacturer’s instructions and western blotting was performed as described previously . Antibodies were listed as follow: P22^phox^ (BosterBio, Pleasanton, CA, USA), P40^phox^ (Boster), Bax (Boster), Bcl2 (Boster), P-MBS (Abcam, Cambridge, UK), T-MBS (Abcam), CD36 (Boster), PPARα (Abcam) and β-actin (Boster).

**PPARα staining in H9C2 cells**

PPARα transcriptional activity is based on the formation of its heterodimers which can translocate into the nucleus and bind to peroxisome proliferator response element (PPRE) in the promoter region of their targets genes. Thus, we detected PPARα nuclear by immunofluorescence in situ. Briefly, Cells were fixed with 4% paraformaldehyde after different treatments at room temperature for 30 min and incubated with PPARα antibody (Abcam) overnight at 4°C, followed by detection with PE-labelled fluorescent secondary antibodies (BD, Franklin Lakes, NJ, USA) for 1 h. Nuclei were counterstained with DAPI (Beyotime) and nuclear PPARα positive cells were quantified by the Image-Pro Plus.

**Reactive oxygen species assay (ROS) and Dihydroethidium (DHE) staining.**

The production of intracellular ROS was analyzed with ROS assay kit (S0033, Beyotime, Shanghai, China) and DHE (DHE, S0063, Beyotime, Shanghai, China) respectively according to the manufacturer’s instructions. Briefly, treated cells or fresh frozen sections were washed with PBS, and then ROS-capturing reagents DCFH-DA or DHE were added and incubated at 37°C in the dark for 30 min and visualized by fluorescence microscopy.

**BODIPY lipids staining and Oil red O.**

BODIPY 493/503 (Invitrogen, Germany) has been used as a stain for neutral lipids and as a tracer for oil and other nonpolar lipids as previously reported. In this study, cultured cells interfered with high glucose and FFA with or without Ex-4 or wy-14643 were stained by BODIPY and then imaged by fluorescence microscopy. Myocardial lipids accumulation in mice heart frozen sections was measured by staining with Oil Red O as previously described .

**Myocardial triglycerides (TG) content detection**

Myocardial triglycerides (TG) content were detected by colorimetric assays according to the introduction of TG detection kit (Nanjing jiancheng bioengineering institute, China). Briefly, myocardial tissue were lysed in ice-cold PBS and then extracted by the addition of 2:1 chloroform/methanol. The dried organic phase was re-suspended in 100% ethanol and analyzed using an enzymantic colorimetric method (GPO-PAP reagent, Rohe Diagnostics).

**Electrophoretic Mobility Shift Assays (EMSA).**

Nuclear extracts were prepared as instructions indicated . Oligonucleotides containing PPAR binding site (forward, 5`-CAA **AAC TAG GTC AAA GGT** **CA**-3`, and reverse, 3’-GTT TTG ATC CAG TTT CCA GT-5’) end-labeled with biotin was purchased from Beyotime (Shanghai, China). Briefly, the biotin-labeled probe was incubated with 10μg nuclear extracts for 30 min at room temperature. The protein-DNA complexes were separated in a 5% nondenaturing PAGE gel. The relative bands intensity was analyzed using Gel pro-analysis software.

**Supplemental reference**

Boldyrev IA, Zhai X, Momsen MM, Brockman HL, Brown RE, Molotkovsky JG (2007). New BODIPY lipid probes for fluorescence studies of membranes. *Journal of lipid research*. **48**, 1518-1532.

Chang G, Zhang D, Liu J, Zhang P, Ye L, Lu K, Duan Q, Zheng A, Qin S (2014). Exenatide protects against hypoxia/reoxygenation-induced apoptosis by improving mitochondrial function in H9c2 cells. *Experimental biology and medicine (Maywood, N.J.)*. **239**, 414-422.

Dai M, Wu L, Wang P, Wen Z, Xu X, Wang DW (2017). CYP2J2 and Its Metabolites EETs Attenuate Insulin Resistance via Regulating Macrophage Polarization in Adipose Tissue. *Scientific reports*. **7**, 46743.

Makia NL, Goldstein JA (2016). CYP2C8 Is a Novel Target of Peroxisome Proliferator-Activated Receptor alpha in Human Liver. *Molecular pharmacology*. **89**, 154-164.

Mori J, Patel VB, Abo Alrob O, Basu R, Altamimi T, Desaulniers J, Wagg CS, Kassiri Z, Lopaschuk GD, Oudit GY (2014). Angiotensin 1-7 ameliorates diabetic cardiomyopathy and diastolic dysfunction in db/db mice by reducing lipotoxicity and inflammation. *Circulation. Heart failure*. **7**, 327-339.

Noyan-Ashraf MH, Shikatani EA, Schuiki I, Mukovozov I, Wu J, Li RK, Volchuk A, Robinson LA, Billia F, Drucker DJ, Husain M (2013). A glucagon-like peptide-1 analog reverses the molecular pathology and cardiac dysfunction of a mouse model of obesity. *Circulation*. **127**, 74-85.

Wang B, Nie J, Wu L, Hu Y, Wen Z, Dong L, Zou MH, Chen C, Wang DW (2017). AMPKalpha2 Protects Against the Development of Heart Failure by Enhancing Mitophagy via PINK1 Phosphorylation. *Circulation research*.

Wang D, Luo P, Wang Y, Li W, Wang C, Sun D, Zhang R, Su T, Ma X, Zeng C, Wang H, Ren J, Cao F (2013). Glucagon-like peptide-1 protects against cardiac microvascular injury in diabetes via a cAMP/PKA/Rho-dependent mechanism. *Diabetes*. **62**, 1697-1708.

Wu L, Zhao F, Dai M, Li H, Chen C, Nie J, Wang P, Wang DW (2017). P2y12 Receptor Promotes Pressure Overload-Induced Cardiac Remodeling via Platelet-Driven Inflammation in Mice. *Hypertension (Dallas, Tex. : 1979)*. **70**, 759-769.

Yang J, Sambandam N, Han X, Gross RW, Courtois M, Kovacs A, Febbraio M, Finck BN, Kelly DP (2007). CD36 deficiency rescues lipotoxic cardiomyopathy. *Circulation research*. **100**, 1208-1217.


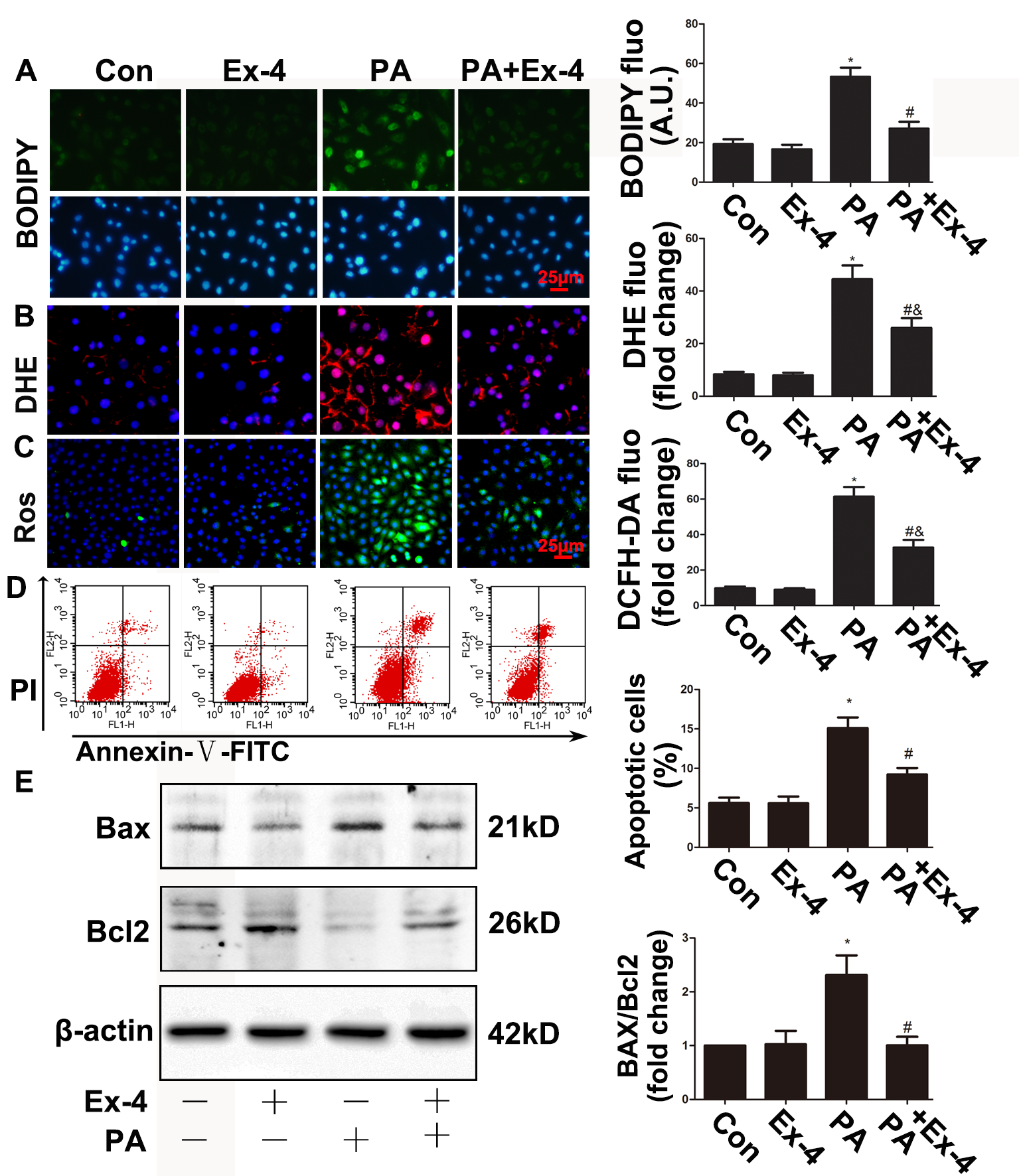


**Figure S1. Exendin-4 attenuated lipid accumulation, oxidative stress, apoptosis and inflammation in palmitic acid-induced H9C2 cells.** (A) Intracellular neutral lipids were traced by BODIBY 493/503 and nucleus was marked by Hoechst 33342. N=6. *P<0.05 vs. control, ^#^P<0.05 vs. PA group. Bar = 25μm. (B-C) Representative images (left) and quantification (right) of intracellular ROS production detected by DCFH-DA and DHE fluorescence. N=6. *P<0.05 vs. control, ^#^P<0.05 vs. PA group, ^&^P<0.05 vs. Ex-4 group. Bar = 25μm. (D) Representative images (left) and quantitation (right) of flow cytometry analysis. N=6. *P<0.05 vs. control, ^#^P<0.05 vs. PA group. (E) Expression of Bax and Bcl2 protein in cell lysis were detected by western blot. N = three independent experiments. *P<0.05 vs. control, ^#^P<0.05 vs. PA group.


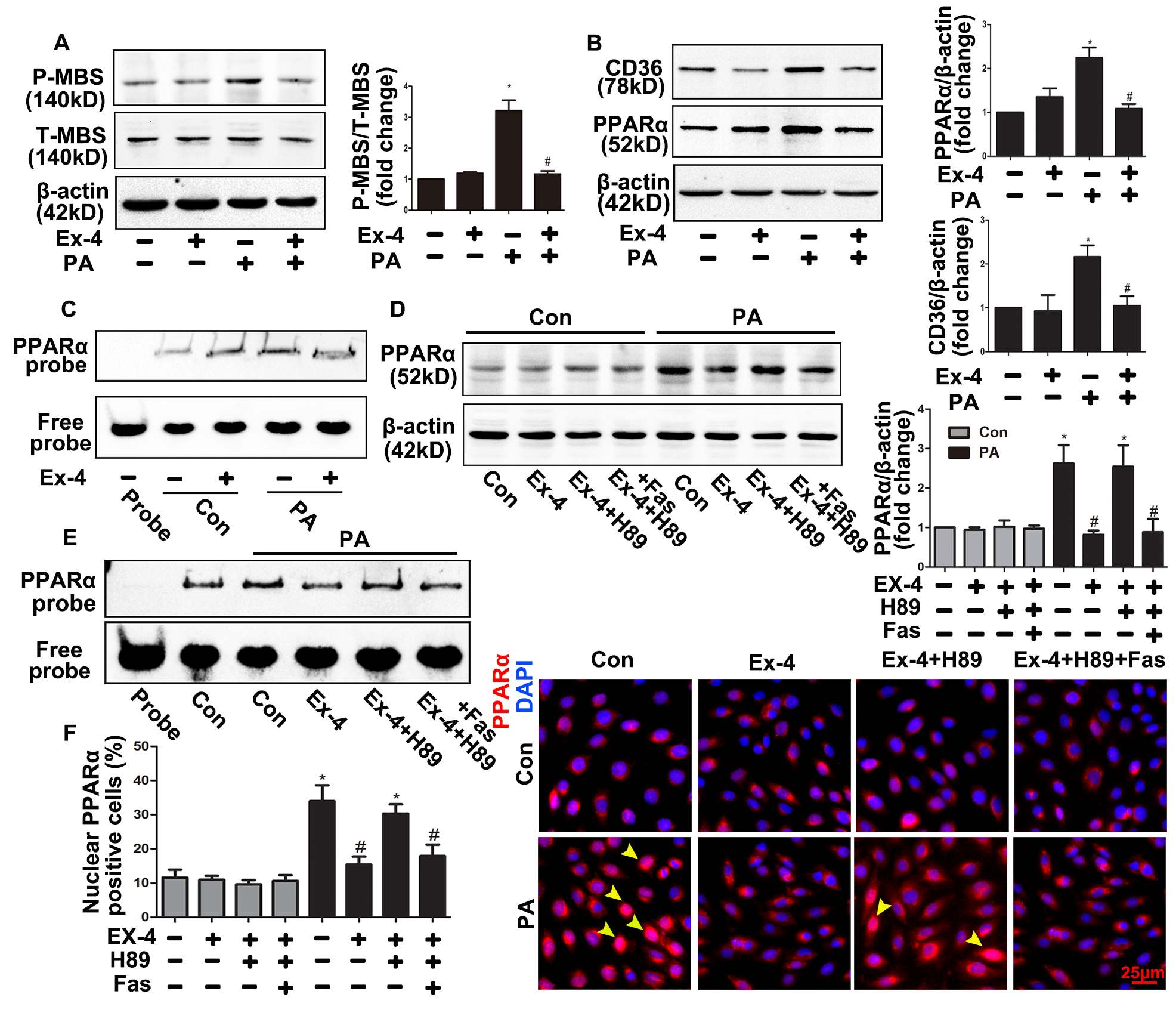


**Figure S2. Exendin-4 inhibited PPAR**α **activation through PKA-ROCK pathway.**

(A-B) Western blot assay for ROCK (A) and PPARα pathways (B) in PA-induced H9C2 cells. *P<0.05 vs. ND, ^#^P<0.05 vs. PA group. (C)The DNA-binding activity of PPARα to PPRE in H9C2 cells treated with PA and Ex-4 was detected by electrophoretic mobility shift assay (EMSA). N=three independent experiments. (D) Expression of PPARα in H9C2 cells treated with PA, Ex-4, H89 and Fasudil. N=Three independent experiments. *P<0.05 vs. control, ^#^P<0.05 vs. PA group. (E) The DNA-binding activity of PPARα to PPRE in H9C2 cells was detected by electrophoretic mobility shift assay (EMSA). N=three independent experiments. (F) Representative PPARα/DAPI staining images for each group and quantification of nuclear PPARα positive cells (yellow arrow). N=6. *P<0.05 vs. control, ^#^P<0.05 vs. PA group. Bar = 25μm.


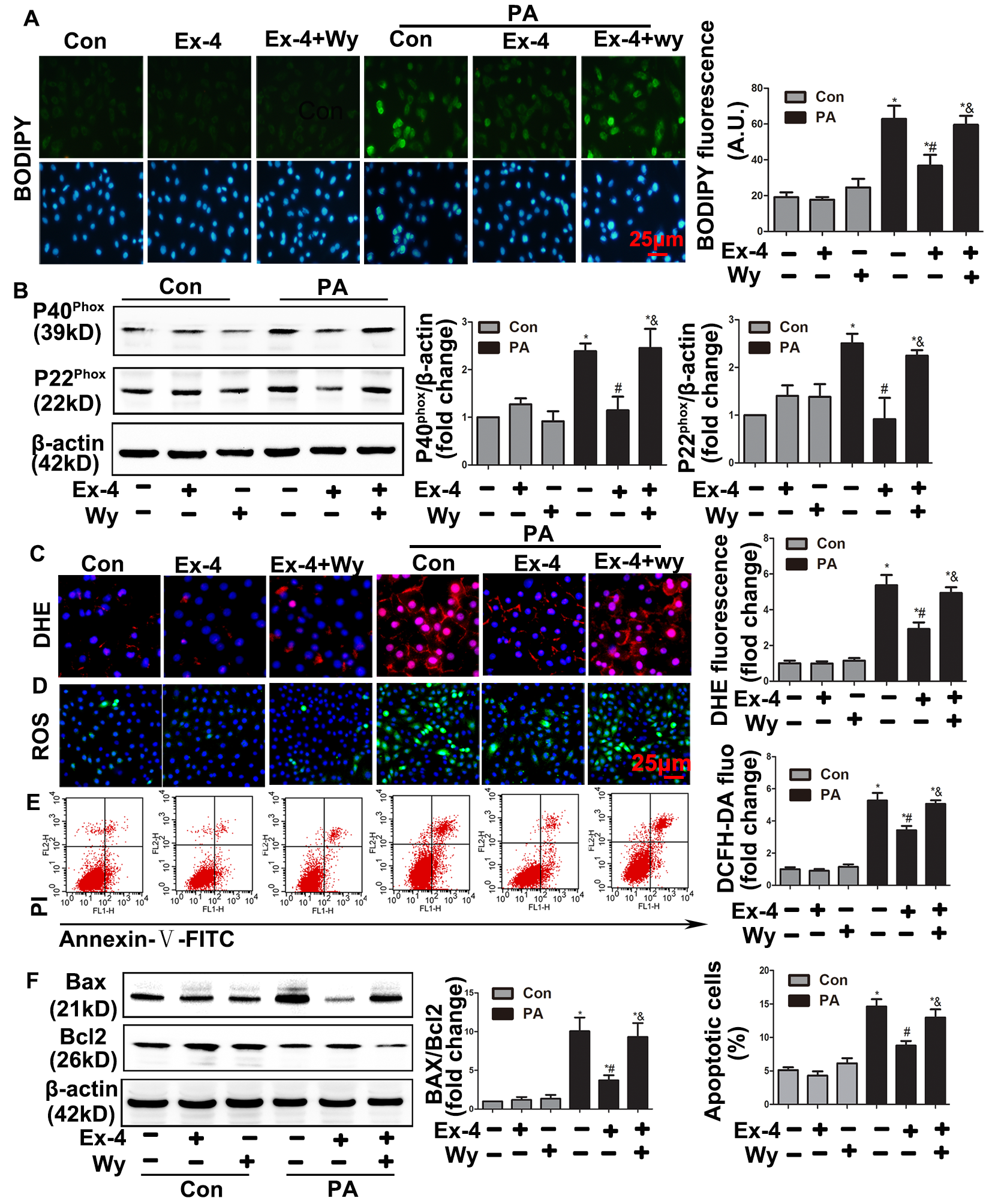


**Figure S3. Exendin-4 mitigated PA-induced lipotoxicity through PPAR**α **pathway in H9C2 cardiomyocyte.** (A) Neutral lipids accumulation in H9C2 was traced by BODIBY 493/503 after treatment with PA, Ex-4 and wy-14643. N=6. *P<0.05 vs. ND, ^#^P<0.05 vs. PA group, ^&^P<0.05 vs. PA+Ex-4 group. (B) Expression of P20^phox^ and P40^phox^ protein detected by western blot. N=three independent experiments. *P<0.05 vs. ND, ^#^P<0.05 vs. PA group, ^&^P<0.05 vs. PA+Ex-4 group. (C-D) Representative images and quantification of intracellular ROS production detected by DCFH-DA and DHE fluorescence. N=7. *P<0.05 vs. ND, ^#^P<0.05 vs. PA group, ^&^P<0.05 vs. PA+Ex-4 group. (E) Representative images (left) and quantitation (right) of flow cytometry analysis. N=8. Mean ± SEM was marked on each image for space limit. (F) Expression of Bax and Bcl2 protein was detected by western blot. N=three independent experiments. *P<0.05 vs. control, ^#^P<0.05 vs. PA group, ^&^P<0.05 vs. PA+Ex-4 group


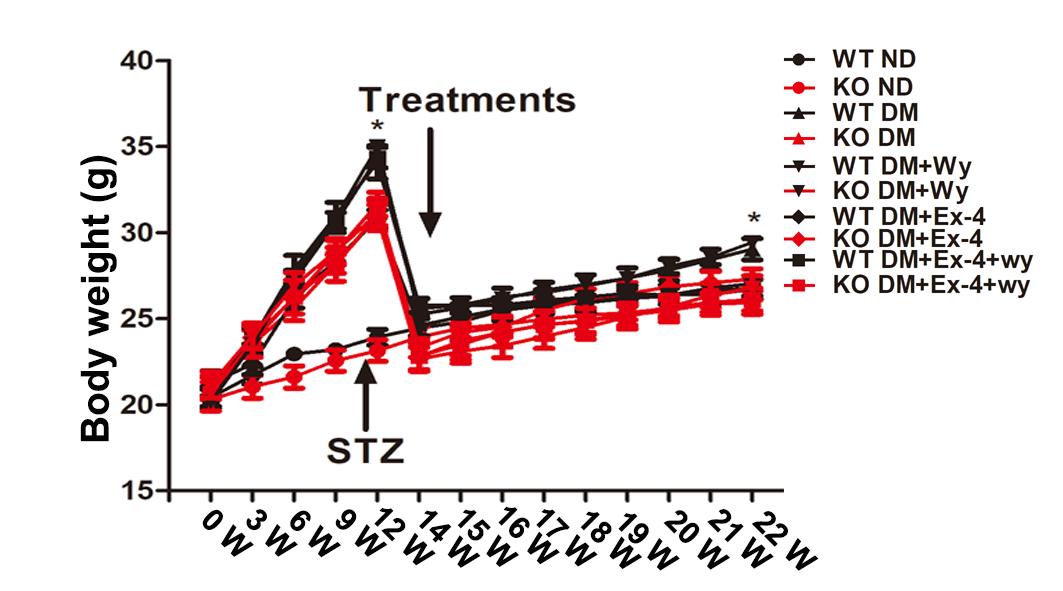


**Figure S4. Body weight of wild-type mice and PPARα knockout mice with different treatments.** *P<0.05 WT DM vs. KO DM N=6-8.


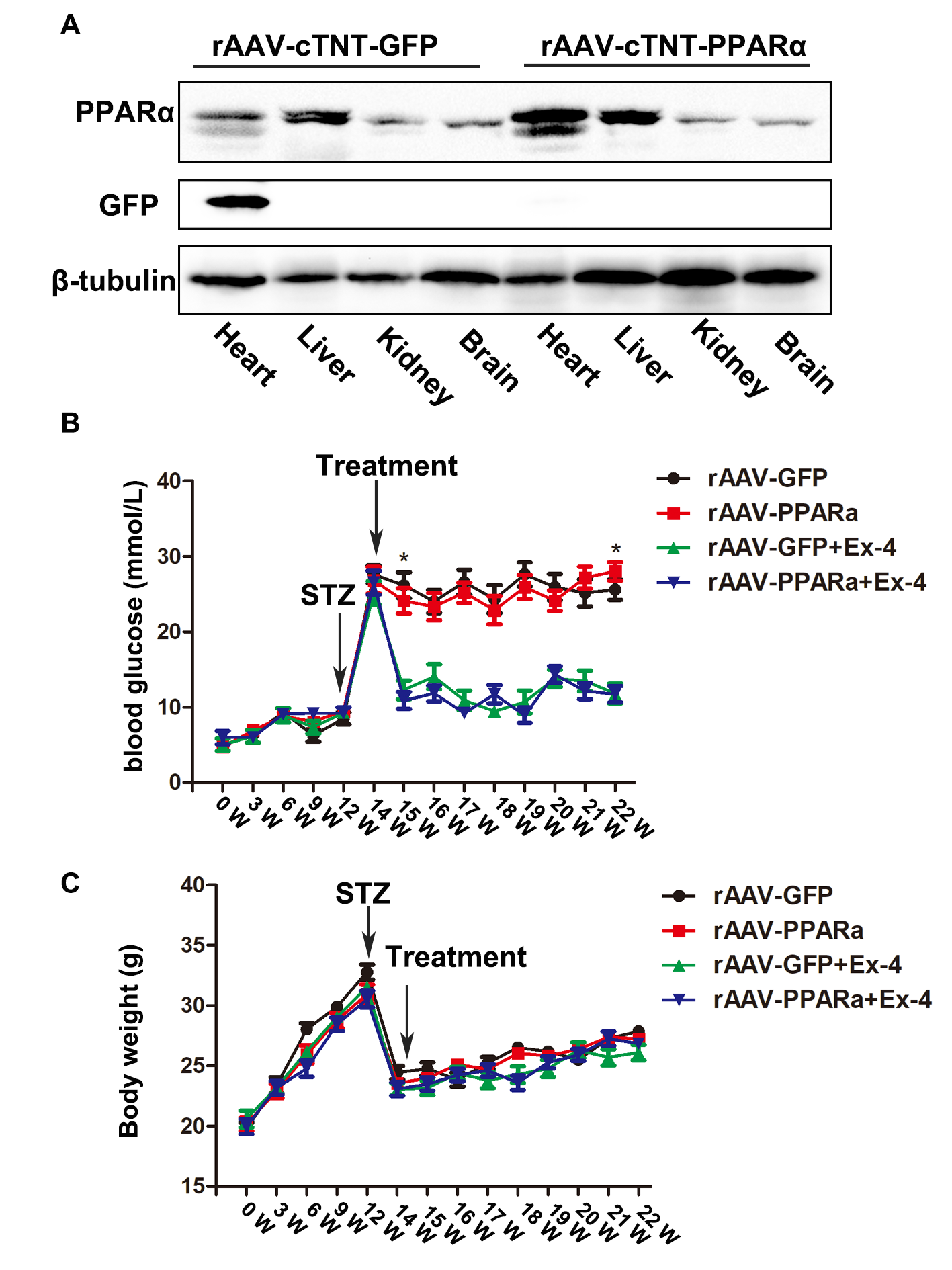


**Figure S5. Cardiac-specific PPARα overexpression mice were successfully built by rAAV-cTNT-PPARα virus injection and their glucose levels and body weights were collected in diabetic state.** (A) Expression of GFP and PPARα protein in mice heart, liver, kidney and brain after rAAV-cTNT-GFP and rAAV-cTNT-PPARα delivery was detected by Western blot. (B) Random blood glucose was monitored at different time point.*P<0.05 vs. rAAV-cTNT-GFP or rAAV-cTNT-PPARα plus Ex-4 treatment. (C) Continuous body weight detection of rAAV-cTNT-GFP and rAAV-cTNT-PPARα mice.

**Table S1. Haemodynamic Parameters and echocardiographic Data of diabetic mice treated with exendin-4, saxasliptin or insulin.**

| Group | ND | DM | DM+Ex-4 | DM+Saxa | DM+insulin |
| --- | --- | --- | --- | --- | --- |
| N | 7 | 8 | 9 | 8 | 8 |
| IVS;d | 0.92±0.05 | 0.99±0.04 | 0.92±0.04 | 0.94±0.34 | 1.00±0.04 |
| IVS;s | 1.19±0.07 | 1.29±0.06 | 1.16±0.06 | 1.17±0.05 | 1.25±0.06 |
| LVID;d | 3.14±0.05 | 3.73±0.09* | 3.43±0.08 | 3.33±0.06^†^ | 3.73±0.11* |
| LVID;s | 2.23±0.14 | 2.76±0.13* | 2.38±0.07 | 2.44±0.05 | 2.65±0.08* |
| LVPW;d | 0.84±0.07 | 0.92±0.03 | 0.81±0.03 | 0.88±0.04 | 0.96±0.04 |
| LVPW;s | 1.09±0.10 | 1.16±0.04 | 1.05±0.06 | 1.10±0.05 | 1.17±0.05 |
| FS (%) | 39.61±1.75 | 23.62±1.41* | 30.4±1.30*^†^ | 30.61±0.77*^†^ | 24.99±1.25*^‡^ |
| HR (BPM) | 348.3±18.01 | 334.6±17.94 | 354.8±23.43 | 337.7±12.2 | 329.2±7.93 |
| Max dP/dt (mmHg/s) | 10817±406.4 | 6815±388.9 | 8386±204 | 8333±324.1 | 7025±411.4 |
| Min dP/dt  (mmHg/s) | -8751±305.7 | -4844±200.9 | -6547±438.6 | -6328±492.3 | -4926±311.2 |
| HR (BPM) | 462±25.14 | 431.2±22.7 | 448.1±22.29 | 421.7±18.31 | 436.7±26.57 |

Max dP/dt, maximal slope of systolic pressure increment; Min dP/dt, minimal slope of diastolic pressure decrement; HR, heart rate; BPM, beat per minute; IVS,d, interventricular septal thickness at diastole; IVS,s, interventricular septal thickness at systole; LVPW,d, LV posterior wall thickness at diastole; LVPW,s, LV posterior wall thickness at systole; LVID,d, LV internal diameter at diastole; LVID,s, LV internal diameter at systole; FS, fractional shortening; Values represent mean ± SEM.

*P<0.05 vs. ND group

^†^P<0.05 vs. DM group

^‡^P<0.05 vs. DM + Ex-4 group and DM + Saxa group.

**Table S2. Haemodynamic Parameters and echocardiographic Data of wildtype (WT) and PPARα^-/-^ (KO) diabetic mice treated with exendin-4 and wy-14643.**

|  | WT ND | KO ND | WT DM | KO DM | WT  DM+Wy | KO  DM+Wy | WT DM+Ex-4 | KO DM+Ex-4 | WT DM+  Ex-4+wy | KO DM+  Ex-4+wy |
| --- | --- | --- | --- | --- | --- | --- | --- | --- | --- | --- |
| N | 6 | 7 | 7 | 8 | 7 | 6 | 8 | 8 | 8 | 7 |
| IVS;d | 0.99±0.07 | 0.99±0.05 | 1.09±0.05 | 1.01±0.13 | 0.98±0.06 | 1.01±0.06 | 1.02±0.06 | 1.01±0.05 | 1.09±0.05 | 1.02±0.03 |
| IVS;s | 1.27±0.06 | 1.26±0.05 | 1.34±0.07 | 1.21±0.16 | 1.21±0.07 | 1.24±0.03 | 1.22±0.05 | 1.23±0.05 | 1.32±0.05 | 1.25±0.04 |
| LVID;d | 2.90±0.18 | 2.96±0.14 | 3.53±0.14* | 3.04±0.07^†^ | 3.80±0.18* | 3.08±0.11 | 3.08±0.07 | 3.06±0.07^†^ | 3.44±0.07* | 3.10±0.07 |
| LVID;s | 2.02±0.10 | 2.05±0.08 | 2.54±0.13* | 2.20±0.08 | 2.99±0.21* | 2.04±0.16 | 2.20±0.08 | 2.19±0.11 | 2.47±0.07* | 2.20±0.08 |
| LVPW;d | 0.95±0.05 | 0.90±0.04 | 1.03±0.05 | 0.91±0.04 | 0.99±0.06 | 0.98±0.10 | 0.94±0.08 | 1.00±0.06 | 1.02±0.03 | 1.01±0.04 |
| LVPW;s | 1.25±0.07 | 1.20±0.03 | 1.28±0.06* | 1.14±0.06 | 1.19±0.05 | 1.18±0.09 | 1.16±0.05 | 1.20±0.06 | 1.3±0.05 | 1.20±0.06 |
| FS | 37.5±2.13 | 38.34±1.35 | 22.79±1.95* | 30.46±1.39^†^ | 19.91±1.39* | 31.21±2.06^†^ | 29.07±1.01* | 31.11±1.51^†^ | 25.74±1.66* | 31.46±1.059^†^ |
| HR (BPM) | 374.0±30.16 | 367.6±24.1 | 346.1±44.36 | 392.8±22.19 | 369.3±11.65 | 353.1±16.4 | 371.7±33.76 | 379.1±26.25 | 385.6±20.89 | 363.9±6.48 |
| Max dP/dt (mmHg/s) | 10320±  247.8 | 10173±  735.2 | 6346±  323.6* | 8014±  367.5*^†^ | 6167±  1015* | 8077±  443.2*^†^ | 8203±  255.3*^†^ | 8168±  261.7*^†^ | 6547±  271.7*^‡^ | 8304±  385.4*^§^ |
| Min dP/dt (mmHg/s) | -7330±  271.9 | -7367±  280.4 | -4580±  191.4* | -6110±  175.7*^†^ | -4898±  746.4* | -6112±  456.6*^†^ | -6138±  221.7*^†^ | -6366±  169.0*^†^ | -4975±  361.6*^‡^ | -6756±  423.8*^§^ |
| HR (BPM) | 468±25.7 | 447.2±33.07 | 420.4±24.61 | 439.3±30.08 | 448±35.58 | 430±34.67 | 445.3±255.3 | 434.8±22.23 | 436.8±21.95 | 466.6±21.17 |

Max dP/dt, maximal slope of systolic pressure increment; Min dP/dt, minimal slope of diastolic pressure decrement; HR, heart rate; BPM, beat per minute; IVS,d, interventricular septal thickness at diastole; IVS,s, interventricular septal thickness at systole; LVPW,d, LV posterior wall thickness at diastole; LVPW,s, LV posterior wall thickness at systole; LVID,d, LV internal diameter at diastole; LVID,s, LV internal diameter at systole; FS, fractional shortening; Values represent mean ±SEM.

*P<0.05 vs. WT ND group

^†^P<0.05 vs. WT DM group

^‡^P<0.05 vs. WT DM+Ex-4 group.

^§^P<0.05 vs. WT DM and WT DM+Ex-4+wy group.

Table S3. **Haemodynamic Parameters and echocardiographic Data of rAAV-cTNT-GFP and rAAV-cTNT-PPARα diabetic mice treated with exendin-4.**

|  | DM  rAAV-GFP | DM rAAV-PPARα | DM rAAV-GFP+Ex-4 | DM rAAV-PPARα+Ex-4 |
| --- | --- | --- | --- | --- |
| N | 8 | 8 | 8 | 8 |
| IVS;d | 1.01±0.03 | 1.03±0.04 | 0.99±0.04 | 1.01±0.03 |
| IVS;s | 1.21±0.04 | 1.27±0.08 | 1.21±0.04 | 1.22±0.03 |
| LVID;d | 3.67±0.14 | 3.64±0.09 | 3.15±0.27 | 3.54±0.18 |
| LVID;s | 2.76±0.14 | 2.81±0.12 | 2.37±0.18 | 2.67±0.21 |
| LVPW;d | 0.98±0.04 | 0.94±0.05 | 0.88±0.09 | 0.92±0.01 |
| LVPW;s | 1.12±0.03 | 1.07±0.06 | 1.03±0.06 | 1.15±0.06 |
| FS | 24.1±0.99 | 19.42±1.48* | 28.84±1.20* | 23.64±0.97^†^ |
| HR (BPM) | 322.6±20 | 354.6±27.5 | 346.4±32.9 | 390.1±26.83 |
| Max dP/dt (mmHg/s) | 6913±178.8 | 5664±180* | 8380±596.5* | 6389±164.1^†^ |
| Min dP/dt (mmHg/s) | -5283±228.4 | -4440±309* | -7109±679.5* | -5411±305.3^†^ |
| HR (BPM) | 370.9±34.28 | 430.3±27.93 | 426.8±42.22 | 445.3±31.35 |

Max dP/dt, maximal slope of systolic pressure increment; Min dP/dt, minimal slope of diastolic pressure decrement; HR, heart rate; BPM, beat per minute; IVS,d, interventricular septal thickness at diastole; IVS,s, interventricular septal thickness at systole; LVPW,d, LV posterior wall thickness at diastole; LVPW,s, LV posterior wall thickness at systole; LVID,d, LV internal diameter at diastole; LVID,s, LV internal diameter at systole; FS, fractional shortening; Values represent mean ± SEM.

*P<0.05 vs. rAAV-GFP DM group,

^†^P<0.05 vs. rAAV-GFP DM + Ex-4 group

**Table S4. Primer sequences for quantitative real-time PCR.**

| **Gene** | **Forward** | **Reverse** |
| --- | --- | --- |
| Nppa-mouse | CGTGCCCCGACCCACGCCAGCATGG | GCCTCCGAGGGCCAGCGAGCAGAGC |
| Collagen1a1-mouse | GAGCGGAGAGTACTGGATCG | TACTCGAACGGGAATCCATC |
| TNF-α-mouse | GGTGACCAGGCTGTCGCTAC | ATCTTTATTTCTCTCAATGACCCGT |
| IL-1β-mouse | TACAAGGAGAAGAAAGTAATGACAA | AGCTTGTTATTGATTTCTATCTTGT |
| GAPDH-mouse | AGGTCGGTGTGAACGGATTTG | TGTAGACCATGTAGTTGAGGTCA |
